# Deep learning methods may not outperform other machine learning methods on analyzing genomic studies

**DOI:** 10.3389/fgene.2022.992070

**Published:** 2022-09-23

**Authors:** Yao Dong, Shaoze Zhou, Li Xing, Yumeng Chen, Ziyu Ren, Yongfeng Dong, Xuekui Zhang

**Affiliations:** ^1^ School of Artifcial Intelligence, Hebei University of Technology, Tianjin, China; ^2^ Department of Mathematics and Statistics, University of Victoria, Victoria, BC, Canada; ^3^ Hebei Province Key Laboratory of Big Data Computing, Tianjin, China; ^4^ Department of Mathematics and Statistics, University of Saskatchewan, Saskatoon, Saskatoon

**Keywords:** deep learning, machine learning, genomic analysis, disease prediction, imbalance data, hit curve

## Abstract

Deep Learning (DL) has been broadly applied to solve big data problems in biomedical fields, which is most successful in image processing. Recently, many DL methods have been applied to analyze genomic studies. However, genomic data usually has too small a sample size to fit a complex network. They do not have common structural patterns like images to utilize pre-trained networks or take advantage of convolution layers. The concern of overusing DL methods motivates us to evaluate DL methods’ performance versus popular non-deep Machine Learning (ML) methods for analyzing genomic data with a wide range of sample sizes. In this paper, we conduct a benchmark study using the UK Biobank data and its many random subsets with different sample sizes. The original UK Biobank data has about 500k participants. Each patient has comprehensive patient characteristics, disease histories, and genomic information, i.e., the genotypes of millions of Single-Nucleotide Polymorphism (SNPs). We are interested in predicting the risk of three lung diseases: asthma, COPD, and lung cancer. There are 205,238 participants have recorded disease outcomes for these three diseases. Five prediction models are investigated in this benchmark study, including three non-deep machine learning methods (Elastic Net, XGBoost, and SVM) and two deep learning methods (DNN and LSTM). Besides the most popular performance metrics, such as the F1-score, we promote the hit curve, a visual tool to describe the performance of predicting rare events. We discovered that DL methods frequently fail to outperform non-deep ML in analyzing genomic data, even in large datasets with over 200k samples. The experiment results suggest not overusing DL methods in genomic studies, even with biobank-level sample sizes. The performance differences between DL and non-deep ML decrease as the sample size of data increases. This suggests when the sample size of data is significant, further increasing sample sizes leads to more performance gain in DL methods. Hence, DL methods could be better if we analyze genomic data bigger than this study.

## 1 Introduction

Machine Learning (ML) has been widely applied in genomic analysis and disease prediction. ML is considered an objective and reproducible method that integrates multiple quantitative variables to improve diagnostic accuracy (Wang et al., 2021). There are many successful applications. In disease prediction, Deberneh and Kim (2021) presented an ML model for predicting the T2D (type 2 diabetes) occurrence in the following year (Y+1) using variables in the current year (Y). The model’s performance proved to be reasonably good at forecasting the occurrence of T2D in the Korean population. Park and Lee (2021) constructed a disease recurrence prediction model using ML techniques. Their study compared the performance of 5 ML models (decision tree, random forest, eXtreme Gradient Boosting [XGBoost], LightGBM, and Stacking models) related to recurrence prediction based on accuracy, and the Decision Tree model showed the best accuracy at 95%. In another study, Hussain et al. (2021) proposed a voting ensemble classifier with 24 features to identify the severity of chronic obstructive pulmonary disease (COPD) patients. Five ML classifiers were applied, namely random forests (RF), support vector machine (SVM), gradient boosting machine (GBM), XGBoost, and K-nearest neighbour (KNN) in their study. These classifiers were trained with a set of 24 features. After that, they combined the results with a soft voting ensemble (SVE) method. The results showed that the SVE classifier outperforms conventional ML-based methods for patients with COPD. In addition, ML-based methods for genetic analysis have also been reported in multiple studies (Rowlands et al., 2019; Placek et al., 2021), such as ML approaches for the prioritization of genomic variants impacting Pre-mRNA splicing; ML suggests the polygenic risk for cognitive dysfunction in amyotrophic lateral sclerosis and so on.

Deep Learning (DL) is a subset of ML, and it goes beyond non-deep ML by creating more complex multi-layered models to mimic how humans function. DL is known to work well in big data applications. Still, DL has been used in disease prediction primarily based on publicly available medical image data, which have common structural patterns to utilize pre-trained networks or take advantage of convolution layers. For example, Chao et al. (2021) presented a DL CVD risk prediction model, which was trained with 30,286 LDCTs from the National Lung Cancer Screening Trial. As a result, the model obtained an area under the curve (AUC) of 0.871 on a separate test set of 2085 subjects and was able to identify patients at high risk of CVD mortality (AUC of 0.768). Zhou et al. (2020) proposed a DL model to classify the HCM genotypes based on a non-enhanced four-chamber view of cine images. Lin et al. (2020) developed and validated a DL algorithm for detecting coronary artery disease (CAD) based on facial photos. Jin et al. (2021) presented a multi-task deep learning approach that allows simultaneous tumour segmentation and response prediction. Their approach to capturing dynamic information in longitudinal images may be broadly used for screening, treatment response evaluation, disease monitoring, and surveillance.

However, compared with image data, genomic data has less structure information to train a DL model. Moreover, building an accurate DL model usually requires immense amounts of data, which is often difficult to find in biological studies with a limited number of participants. Therefore, we are motivated to investigate the effectiveness of DL in genomic analysis and the amount of genomic sample size fitting for the DL model.

Our study explores and compares three non-deep ML and two DL methods in genomic analysis, including elastic net, XGBoost, SVM, long short-term memory (LSTM), and deep neural network (DNN). These methods are applied to the UK Biobank study, which includes a wide array of genotypic and phenotypic information from 502,524 participants. Coupled with the current impact of COVID-19, lung diseases have attracted widespread attention. We choose three specific lung diseases from UK Biobank, combined with SNPs and other relevant covariates to build prediction models with these five typical non-deep ML and DL algorithms. Large-scale computation works are conducted using high-performance computing servers provided by Compute Canada. To investigate how DL and non-deep ML methods perform in genomic analysis on various sample sizes, we generate random subsets of original data with 10 different levels of sample size and evaluate the prediction performance of each method using multiple metrics, including F1 score, precision, recall, and the hit curve. Besides comparing DL and non-deep ML methods, we also investigated the relation between performance change and other important factors, such as sample sizes increase and the imbalanced ratio (defined as the proportion of samples in the number of a control group to the number of case group (Sun et al., 2019).

The rest of the paper is organized as follows. Section 2 provides detailed processing and summary statistics of the dataset from UK Biobank, and five DL and non-deep ML methods are discussed in detail. In Section 3, experiment results are presented and compared. Concluding remarks are given in Section 4.

## 2 Data and methods

**FIGURE 1:**
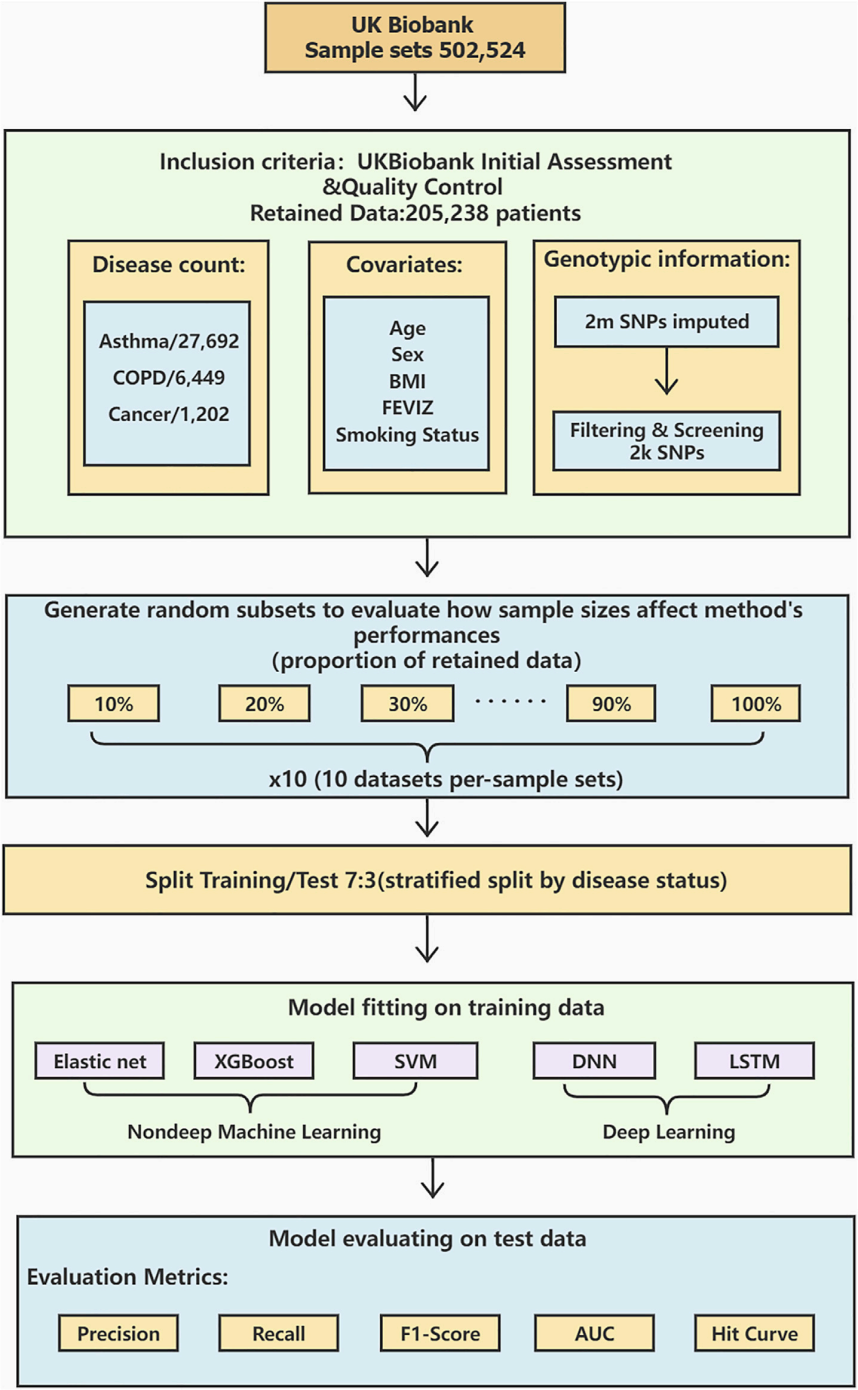
A workflow diagram of the study process. We perform data preprocessing on 502,524 sample sets from UK Biobank. After the initial assessment and quality control, the data is retained for 205,238 cases with detailed procedures in. There are 27,692 asthma cases, 6,449 COPD cases, and 1,202 lung cancer cases. Age, sex, BMI, FEVIZ, and smoking status are covariates. 2,000 SNPs are retained after filtering and screening the original 2 million SNPs. The retained dataset was divided into ten subsets per-sample sets from 10 to 100%. We split the data by disease status into 70% as training and 30% as testing sets. This study uses three non-deep ML models (Elastic net, XGBoost, and SVM) and two DL models (DNN and LSTM) to construct the prediction models. Finally, the model performance is evaluated by the metrics, such as precision, recall, F1-score, AUC, and hit curve.

The workflow diagram is shown in Figure 1.

### 2.1 Data

With the rapid spread of COVID-19, lung diseases have attracted widespread social attention. It was suggested that the presence of lung diseases, in general, may contribute to severe COVID-19 symptoms. About 600 million people have asthma, and lung cancer and COPD are the first and the third leading cause of death worldwide. Genetic variants such as single nucleotide polymorphisms (SNPs) have been focused on in lung disease research.

The dataset we use in our study is the release of the 2018 UK Biobank. The original dataset has collected a wide array of phenotypic and phenotypic information from 502,524 participants. We only select three specific lung diseases (i.e. asthma, COPD, and lung cancer), combined with participants’ SNPs, sex, body mass index (BMI), age, smoking status, and Z-score of the forced expiratory volume in one second (FEV1Z).

#### 2.1.1 Genotype and quality control procedure

Quality control and imputation were performed centrally by UK Biobank. We exclude the following participants from our analyses: 1) participants not of white British ancestry either by self-report or principal component analysis conducted by UK Biobank, 2) participants with more than 10% missing genotype data, 3) participants with putative sex-chromosome aneuploidy, 4) participants where the self-reported sex does not match the genetically-inferred sex, 5) participants that UK Biobank has flagged for having high heterozygosity/missingness and 6) participants with at least ten putative 3rd-degree relatives. Further, we remove SNPs with imputation information score <0.1 , minor allele frequency <0.001 , more than 5% missing genotype data, p-value $<10^{-6}$ in the Hardy-Weinberg Equilibrium test, and SNPs that fail UK Biobank quality control in at least one batch. After sample filtering and SNP screening, we are left with a sample size of 205,238 participants and 2,000 SNPs.

#### 2.1.2 Data statistics

The average age of subjects is 56.5 years, with an age range of 40–69 and a sex ratio (females/males) of 1.35. The selected features are BMI, sex, age, Smoking status, FEV1Z, and 2,000 SNPs information. The summary of data is shown in Table 1. To explore the model performance and the prediction effect of DL and non-deep ML in the case of large and small data, we randomly generate ten subsets from 10 to 100% and repeat it ten times. The detailed subset information is shown in the Supplementary Material (Supplementary Tables S1–S4).

**TABLE 1:** Descriptive statistics of the dataset. This table gives the relationships between smoking status and other covariates, i.e., age, sex, BMI, FEV1Z score, asthma status, COPD status, and lung cancer status.

Covariates	Never smoked	Previously smoked	Currently smokes
Age			
< 55 years	47,137 (42.1%)	22,112 (29.3%)	8,269 (46.6%)
≥55 years	64,826 (57.9%)	53,414 (70.7%)	9,480 (53.4%)
Sex			
Male	69,300 (61.9%)	39,670 (52.5%)	8,912 (50.2%)
Female	42,663 (38.1%)	35,856 (47.5%)	8,837 (49.8%)
BMI_mean	27.00 (±4.67)	27.83 (±4.68)	26.93 (±4.65)
FEV1Z_mean	0.31 (±1.05)	0.44 (±1.10)	0.85 (±1.17)
Asthmastatus	15,110 (13.5%)	10,343 (13.7%)	2,239 (12.6%)
COPDstatus	1,350 (1.2%)	3,338 (4.4%)	1,761 (9.9%)
Cancerstatus	185 (0.17%)	627 (0.83%)	390 (2.2%)

### 2.2 Methods

#### 2.2.1 Elastic net

In general, the elastic net is the regularized linear regression method (Zou and Hastie, 2005). It is a middle ground between ridge regression and lasso regression. The penalty term is a simple mix of ridge and lasso’s penalties, and the mix ratio can be controlled. The estimates from the elastic net method are defined by

$$\hat{\beta }=\arg\underset{\beta }{\min}\left(\|y-X\beta {\|}^{2}+\lambda_{2}\|\beta {\|}_{2}^{2}+\lambda_{1}\|\beta {\|}_{1}\right),$$ (1)

where *λ*_1_‖*β*‖_1_ and $\lambda_{2}\|\beta {\|}_{2}^{2}$ are the *L*_1_ norm and *L*_2_ norm, respectively, *y* is the response variable vector, and *X* is covariates vector. The relationship between *λ*_1_ and *λ*_2_ can be written as

$$\lambda_{1}=\alpha \lambda ,$$ (2)

and

$$\lambda_{2}=\frac{\left(1-\alpha \right)}{2}\lambda .$$ (3)

When the mix ratio *α* approaches 0, the elastic net is equivalent to ridge regression, and as the ratio *α* goes to 1, it is equal to lasso regression. As a result of balancing the L1 norm and L2 norm, the computational cost of the elastic net is expensive. However, it reduces the impact of different features while not eliminating all of the features to improve the model performance.

In this study, Elastic net models are implemented by R. The parameters *α* and *λ* are tuned and chosen by function cv. glmnet ().

#### 2.2.2 XGBoost

XGBoost is an optimized distributed gradient boosting library designed to be efficient, flexible, and portable. It implements the algorithms in the Gradient Boosting framework, which integrates many weak classifiers to form a strong classifier (Ma et al., 2021). The weak classifiers compensate each other to improve the performance of the strong classifier.

Unlike the traditional integrated decision tree algorithm, XGBoost adds a regular term in the loss function to control the complexity of the model while preventing the model from overfitting. The objective function is defined by

$$F\left(x\right)=\overset{n}{\underset{i=1}{\sum }}l\left(y_{i},\hat{y_{i}}\right)+\overset{K}{\underset{k=1}{\sum }}\Omega \left(f_{k}\right),$$ (4)

where $l\left(y_{i},\hat{y_{i}}\right)$ is the model’s loss function, Ω(*f*_*k*_) is the regular term, *n* is the number of samples, and *K* is the number of the CART tree. After that, a second-order Taylor expansion approximation is applied to the loss function, and the objective function is optimized to approach the actual value and improve the prediction accuracy.

GridSearchCV function is used to find the optimal parameters. The parameter max_depth of the XGBoost model is set to 5. The larger the max_depth, the more specific and local samples the model learns. The min_child_weight determines the minimum sum of instance weight needed in a child, and its value is 4. The parameter subsample is 0.8, which controls the proportion of random samples for each tree. The parameter colsample_bytree is used to manage the percentage of columns sampled per randomly sampled tree (each column is a feature), and its value is 0.8. The objective parameter defines the loss function that needs to be minimized. Reg_alpha and reg_lambada are the L1 regularization terms of the weights and the L2 regularization terms of the weights, respectively. These two parameters help reduce overfitting, and their values are 60 and 2, respectively.

#### 2.2.3 SVM

SVM is a supervised learning algorithm. The learning strategy uses supporting vectors and margins to find the optimal segmentation hyperplane to classify the data (Fan et al., 2021). SVM can be used for classification and regression analysis. As a training algorithm, SVM has a highly accurate and strong generalization ability.

This study uses the LinearSVC module in SVM. LinearSVC implements a linear classification support vector machine and can choose a variety of penalty parameters and loss functions. Normalization also works well when the number of training set instances is large.

We add the regularization term L1 norm to reduce the impact of overfitting. The parameter C of the LinearSVC model is 1.0.

#### 2.2.4 LSTM

LSTM is a recurrent neural network (RNN). It can solve the problem of gradient disappearance and gradient explosion in traditional RNN. LSTM consists of a forget gate, an input gate, and an output gate (Elsheikh et al., 2021). The input vector and output vector of the hidden layer of LSTM are *x*_*t*_ and *h*_*t*_, and the forward propagation process can be used in Equations 5-9.

The input gate is mainly used to control how many values of the current input will flow directly to a memory unit, defined as follows:

$$i_{t}=\sigma \left(W_{x_{i}}x_{t}+W_{h_{i}}h_{t-1}+b_{i}\right).$$ (5)

The forget gate is an essential component of the LSTM memory cell, which controls the retention and forgetting of information to avoid gradient disappearance and gradient explosion caused by the backward propagation of gradients over time. The value of the forget gate *f*_*t*_ and the value of the memory cell *c*_*t*_ are expressed as:

$$f_{t}=\sigma \left(W_{x_{f}}x_{t}+W_{h_{f}}h_{t-1}+b_{f}\right)$$ (6)

$$c_{t}=f_{t}⊗c_{t-1}+i_{t}⊗\tanh\left(W_{x_{c}}x_{t}+W_{h_{c}}h_{t-1}+b_{c}\right).$$ (7)

The role of the output gate is to effectively control the effect of a memory processing unit on the input and output values in these messages. The value of the output gate *o*_*t*_ and the output *h*_*t*_ of LSTM at moment *t* are expressed as:

$$o_{t}=\sigma \left(W_{x_{o}}x_{t}+W_{h_{o}}h_{t-1}+b_{o}\right)$$ (8)

$$h_{t}=o_{t}⊗\tanh\left(c_{t}\right).$$ (9)

We construct a network structure with three LSTM layers and one dense layer and use sigmoid as the activation function and binary cross-entropy as the loss function. We set batch size and epoch as 128 and 100, respectively, and the learning rate is 0.0005.

#### 2.2.5 DNN

A deep neural network (DNN) is a framework of deep learning. It is a neural network with at least one hidden layer (Ye et al., 2021), which can also be called a multi-layer perceptron.

For the DNN model, We divide it into the input layer, hidden layer, and output layer. Since we are exploring the classification and prediction of these three diseases, we choose *binary*_*c*_ *rossentropy* as our loss function. Secondly, we put three total connection layers into the hidden layer. The number of neurons in the hidden layer is set to 64. Each neuron in the top connection layer is fully connected with all neurons in the previous layer, which can integrate the local information with category differentiation in each layer. To improve the network performance of DNN, we applied the ReLU function to the activation function of each neuron.

Meanwhile, we found through experiments that when the batch size was set to 128, the model’s accuracy could be effectively improved, and the model could converge more accurately towards the direction where the extreme value was. Moreover, when the epoch was 200 iterations, the training results tended to be stable basically. Although the model performance is improved, it is more prone to overfitting due to many parameters. Therefore, we added a regularization term L1 norm to constrain training parameters by adding a penalty norm for training parameters to the loss function to prevent model overfitting.

## 3 Results

In this study, five disease prediction models based on non-deep ML and DL models, i.e. elastic net, XGBoost, SVM, LSTM, and DNN, are constructed. The original dataset is randomly selected into ten sets of 10–100% datasets (shown in Supplementary Tables S1–S4). In the modelling process, we perform ten cross-validations on each set of 10–100% datasets to find the optimal threshold for prediction and apply it to the test set. Finally, the mean and the standard deviation of the accumulated AUC, precision, recall, F1-score values, and hit curve plot are used as evaluation metrics. The detailed statistics are described in Supplementary Material (Supplementary Tables S5–S7).

**FIGURE 2:**
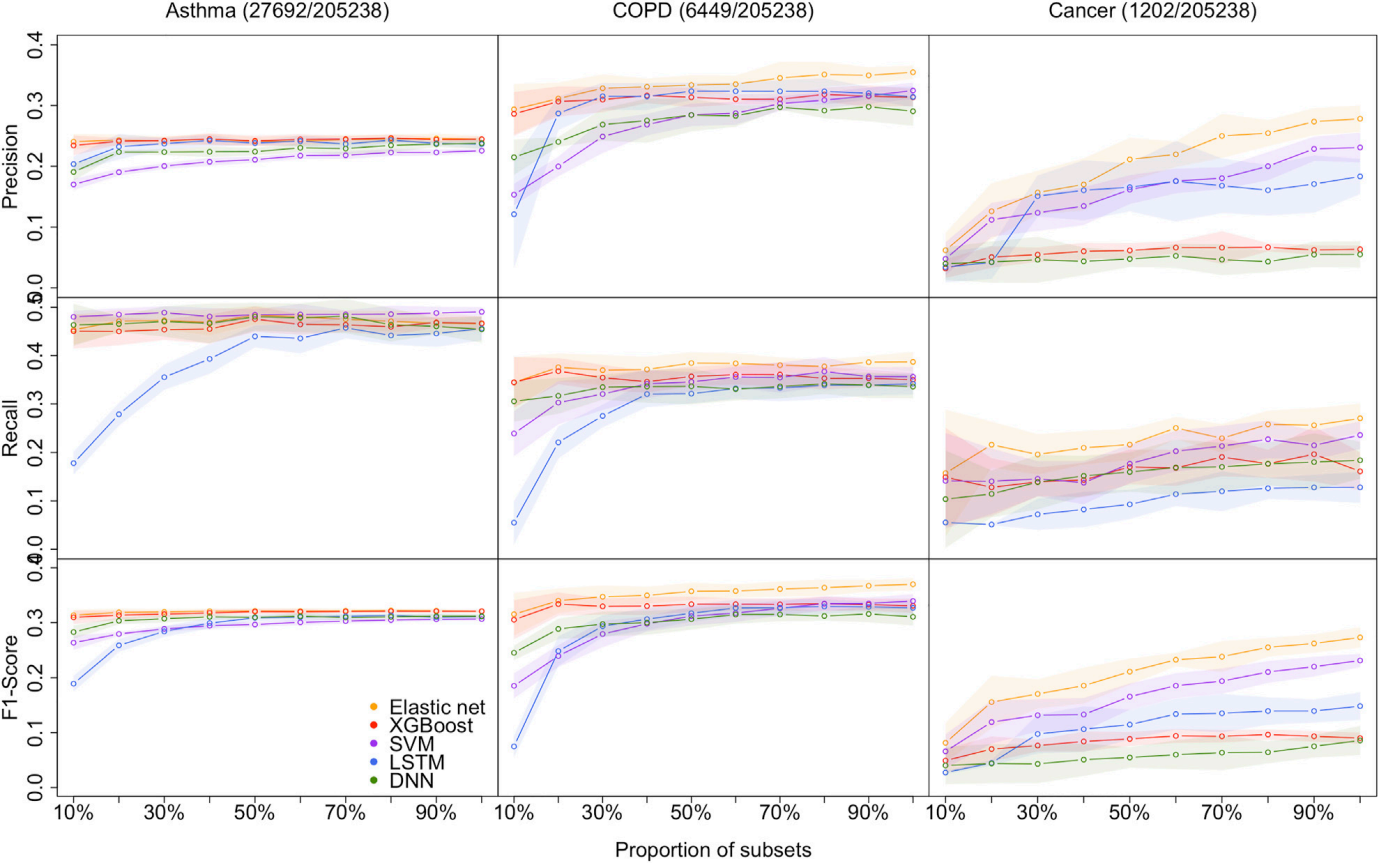
Models performance of the 5 methods with the 10 different sample size for predicting asthma, COPD, and lung cancer, respectively. Performances are shown by precision, recall, and F1-score. the shaded parts are the 1 standard error confidence bounds.

As shown in Figure 2, the proposed models are evaluated by the standard metrics of precision, recall, and F1-score, and an increasing trend is generally discovered. Precision is the proportion of positive predictions that are actually correct. Recall is the proportion of actual patients identified correctly. The F1-score is the harmonic mean of precision and recall, and is often used to interpret imbalanced data. However, AUC is not sensitive to imbalanced data (its results are shown in the Supplementary Material [Supplementary Tables S5–S7])). Hence, we are more interested in precision, recall, and F1-score due to the imbalanced data structure.

### 3.1 Performance on small-sized datasets

**Asthma status prediction.** For the 10% dataset, the highest precision and F1-score are 0.2404 (±0.0127) and 0.3135 (±0.0098), respectively, obtained by the elastic net model. SVM beats other models’ recall value, which is 0.4800 (±0.0126). LSTM has the lowest performance on recall and F1-score, which are 0.1780 (±0.0234) and 0.1891 (±0.0142), respectively. The lowest precision value generated from SVM is 0.1701 (±0.0092). For the 20% dataset, LSTM significantly improved recall and F1-score, but still lower than the other four models. Despite that, the performances of all models remained the same.

**COPD status prediction.** For the 10% dataset, elastic net models’ results are better than that of other models. Its precision, recall, and F1-score are 0.2938 (±0.0415), 0.3446 (±0.0524), and 0.3153 (±0.0386), respectively. The results of XGBoost are very close to those of the elastic net model. However, LSTM has poor performance in this case, and its precision, recall, and F1-score are 0.1210 (±0.0886), 0.0550 (±0.0443), and 0.0749 (±0.0191), respectively. For the 20% dataset, the optimum values of each indicator are also derived from the elastic net. LSTM has a decent improvement in precision performance. And its precision is 0.2871 (±0.0274), while the elastic net has precision value of 0.3115 (±0.0264).

**Cancer status prediction.** All metrics of two DL models underperform that of three non-deep ML models for 10 and 20% datasets. The top F1-score of the two DL models is 0.0402 for the 10% dataset, which is evaluated from the DNN model, whereas the lowest F1-score from non-deep ML methods (XGBoost) is 0.0088 higher.

In summary, it is clear that on a small dataset, the performance of non-deep ML models is superior to that of DL models.

### 3.2 Overall model performance on DL and non-deep ML

As the size of the dataset increases, the overall model performances increase, and the gap between non-deep ML and DL decreases.

**Asthma status prediction.** The F1-score of elastic net, XGBoost, SVM, LSTM, and DNN for 50% dataset are 0.3214 (±0.0047), 0.3201 (±0.0047), 0.2966 (±0.0043), 0.3088 (±0.0060), and 0.3098 (±0.0032), respectively. As the data volume rises to 100%, the performances of the five models do not change a lot.

**COPD status prediction.** When the dataset size increases to 50%, LSTM improves its performance rapidly. The F1-score of LSTM has grown three times from 0.0749 (±0.0191) to 0.3171 (±0.0154). When the dataset size expands from 50 to 100%, the optimal F1-score is 0.3699 (±0.0110) from the elastic net. The F1-scores of XGBoost, SVM, LSTM and DNN become 0.3307 (±0.0130), 0.3394 (±0.0125), 0.3269 (±0.0145), and 0.3106 (±0.0157), respectively.

**Cancer status prediction.** On 50% of the dataset, the performance of all five models has improved. As the dataset grows to 100%, all models’ performances are still climbing up.

In summary, DL models do not outperform non-deep ML models, even in extensive data with over 200k samples. The performance of all models improves when the sample size increases. The performance differences between DL and non-deep ML decrease as the sample size of data increases.

### 3.3 Impact of imbalanced data structure

In this study, the datasets are imbalanced, and the imbalanced rates (Control/Case) for asthma, COPD, and lung cancer are 6.5:1, 30.8:1, and 169.6:1, respectively. Model performances on cancer prediction are the lowest since the cancer dataset structure is highly imbalanced. For example, the F1-score of DNN for the 50% dataset is 0.3098 (±0.0032) for predicting asthma status, whereas it is 0.0547 (±0.0187) for predicting cancer status. Moreover, as the imbalanced rate increases, the confidence bands are getting wider. For instance, the width of the confidence band of XGBoost’s F1-score for the 100% dataset is 0.0058 for predicting asthma; in contrast, it is 0.0202 for predicting lung cancer.

### 3.4 Promote hit curve as a particular visual tool

**FIGURE 3:**
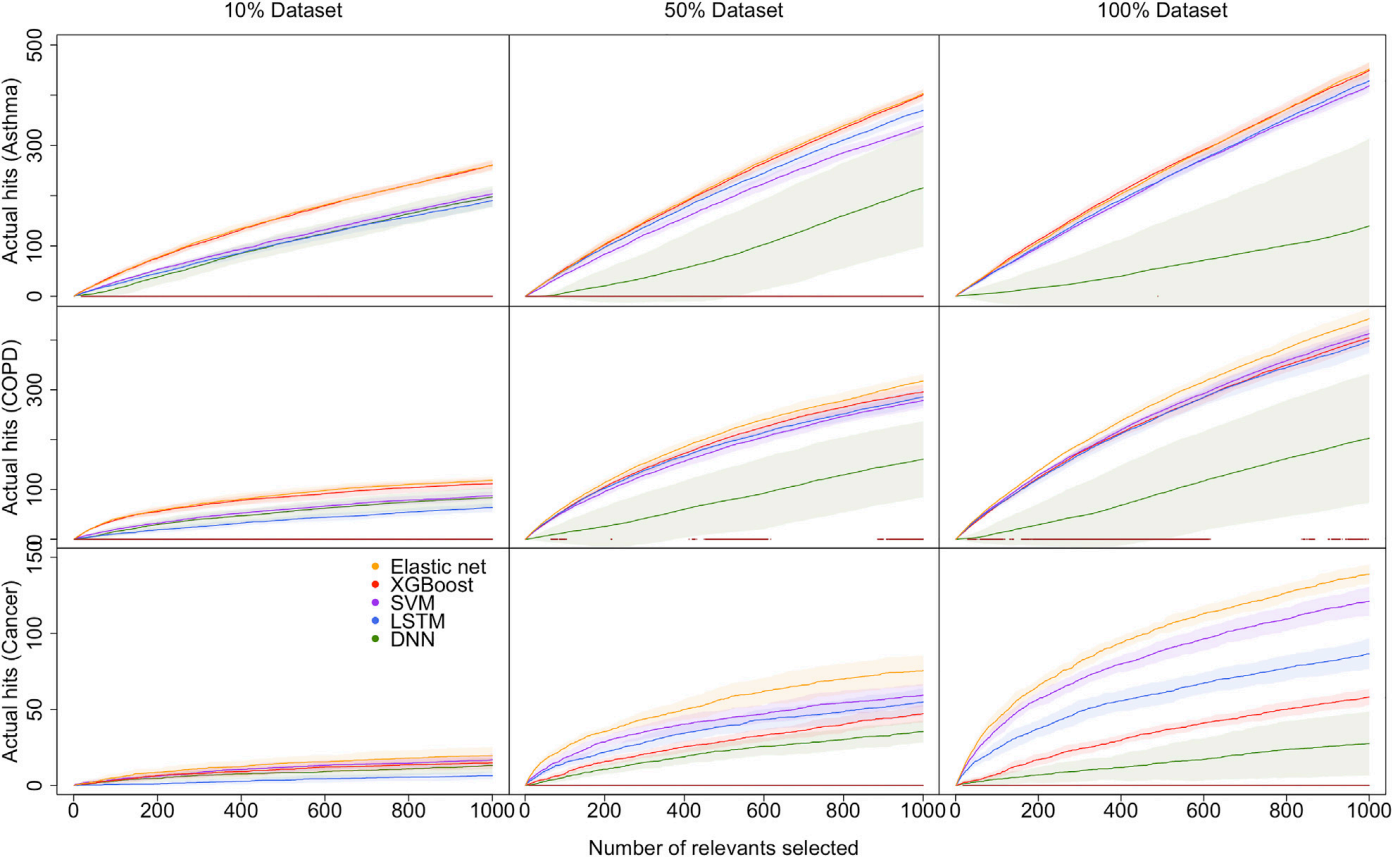
Hit curve graphs of AsthmaStatus, COPDStatus and CancerStatus classification by five models on 10–100% data sets. The x-axis represents the number of test subjects we selected by sorting the estimated probability up to down. The y-axis of the hit curve chart represents the number of subjects with certain conditions which are correctly diagnosed in the test set. The point (*m*_1_, *m*_2_) indicates there are *m*_2_ patients in the first *m*_1_ selected subjects are correctly predicted as diseased. The curves show the average hit curves of five models, and the shaded area denotes the confidence bounds constructed using 10-fold cross-validation (i.e. ± one standard error). The brown bar at the bottom means non-deep ML models are significantly better than DL models.

To summarize all the metric results we have found, a hit curve is promoted as a particular visual tool to compare the prediction models. In a biomedical study, it is impossible for a prediction model to accurately predict all cases, and a model can be effective without necessarily accurately predicting all cases. For example, in our research, a prediction model is considered to be doing an excellent job if it can choose a relatively small number of subjects, and correctly label the majority of the condition group. Therefore, hit curve is used to prioritize case. In this situation, cases with the largest prediction probabilities are chosen first. As we select cases according to the prediction probabilities, a “hit” occurs whenever the case is a success (people we selected are in a certain disease condition). Say we choose *m*_1_ subjects and *m*_2_ are diseased, and we can visually assess a prediction model by plotting *m*_2_ against *m*_1_, a so-called hit curve. A good prediction model will have *m*_2_ increasing rapidly with *m*_1_, as shown in Figure 3 (only the hit curve plots for 10, 50, and 100% of the dataset are shown here, and the result plots for the remaining percentage of the dataset are visible in the Supplementary Material [Supplementary Figures S1–S30]).

The elastic net curve and XGBoost curve are nearly identical, but they cross over each other at some points and are significantly higher than the others in predicting Asthma and COPD. For lung cancer condition prediction, XGBoost does not maintain a good performance. However, elastic net and SVM models are still superior to the LSTM model. DNN model is inferior to other models in all cases. Therefore, evidence supports that DL models often cannot overperform non-deep ML models. The brown bar appears in the 10 and 50% datasets on predicting asthma conditions. However, there are no brown bars in the 100% dataset plot. It implies the performance gap between DL and non-deep ML decreases as the sample size increases. And the difference will be trivial when the data sample size is as large as the biobank level. However, it is difficult to obtain such a large dataset. Hence, DL models often underperform non-deep ML models.

With lung cancer data’s highly imbalanced data structure, none of the five models perform well when the data sample size is small. Their shaded areas are relatively broad, making the difference hard to tell. As the data sample size increases, their hit curves increase with different slopes. As a consequence, the performance differences become substantial. In other words, imbalanced data is also called weighted data. The effective sample size of a weighted data is smaller than its original sample size. It is almost impossible to evaluate those five models’ performances due to a lack of a sufficient sample size. As the effective sample size gradually increases, the model performance differences become apparent. However, if the effective sample size reaches a certain large amount, the differences among all models are not significant again.

## 4 Discussion

This study evaluated the potential of DL models (DNN and LSTM) in predicting asthma, COPD, and lung cancer with various sample sizes from the UK Biobank dataset, compared with non-deep ML models (elastic net, XGBoost and SVM). Besides the most popular performance metrics, such as the F1-score, the hit curve, as a particular visual tool, is promoted to describe the performance of predicting rare events. The results suggest that we should not apply DL methods in most genomic studies, unless we have data with biobank-level sample sizes. We conclude not recommending standard deep learning methods for genomic studies based on the following two facts we observed in our study. First, the prediction performances of non-deep machine learning methods vastly outperform deep learning methods in small datasets (e.g., 10 and 20% random subsets of UK Biobank). Second, we observed that deep learning could not outperform non-deep methods in huge data like the entire cohort of UK Biobank (500k participants), although increasing sample size leads to the improvement of the deep learning method’s performance, and its improvement is faster than non-deep methods. Therefore, we need more data than UK Biobank to prefer deep learning methods. However, the sample sizes of most publicly available genomic data cannot meet this requirement, which range from tens to few thousands.

Although deep learning methods achieved outstanding performance in image, video, and natural language analysis, we found their performance is not attractive in analyzing genomic studies. We believe this is the result of two characteristics of genomic data: 1) genomic data typically has small sample sizes to fit a complex network; and 2) genomic data lacks common structural patterns like images to use pre-trained networks or take advantage of convolution layers.

Besides comparing deep learning methods with non-deep methods, the following are other important messages we learned from this study and would like to share with the audience.

We found that cancer status is much harder to predict than the other two diseases. The results show that the uneven data structure also affects the model’s performance. The control/case ratio is 6.5:1 for asthma, 30.8:1 for COPD, and 169.6:1 for lung cancer, respectively. We notice that all three disease conditions are imbalanced, and the imbalanced ratio of lung cancer conditions is particularly extreme, which leads to model overfitting and underperforming prediction. Therefore, the imbalanced rate between cases and controls is also a critical influencing factor. Although we operate by regulation, rare events are harder to predict. We would do the data augmentation to prevent the imbalance problem in the future.

Our predictions of disease status are based on genomic information but not the specific diagnostic tests of related diseases. Therefore, we don’t expect high accuracy in the predictions. This prediction aims to segment the patients by their predicted risk of conditions and manage them differently (e.g., following up with a different visit frequency or using follow-up disease-specific diagnostic tests).

There are two types of classification mistakes: 1) incorrectly labeling a patient as low-risk or healthy; and 2) incorrectly labeling a healthy individual as a patient or high-risk. In our case, the first type of mistake is much more harmful than the second type. Follow-up diagnosis can fix the second mistake. The first mistake may cause a delay in treatment, while the timing of treatment can be the most critical factor in treating diseases like cancer. These two types of mistakes can be summarised by precision and recall, respectively. The most popular metric, F1-score, is the harmonic average of precision and recall, which regards these two prediction mistakes as costing equally. Fn-score can weigh two types of mistakes using user-defined weights. However, it is not easy to define weights objectively. Hence, we introduced our preferred metric, the hit curve, for rare event detection, which focus on detecting true positive rate. Different points on the curve correspond to different decision rules about who should be labelled as patients. Users can compare many decision rules between the two methods using their hit curves. Users can also use this visual tool to decide which decision rule is best (subjectively).

## Data Availability

The codes are available at (https://github.com/hebutdy/Evaluation-on-GS). The datasets for this study can be found in the UK Biobank (https://www.ukbiobank.ac.uk/).
