# Supplementary material for: Deep learning methods may not outperform other machine learning methods on analyzing genomic studies
Source: Front Genet. 2022 Sep 23;13:992070. doi: 10.3389/fgene.2022.992070 (PMC9537734; doi:10.3389/fgene.2022.992070)
Supplement: Supplementary file 1 [file DataSheet1.PDF]

Supplementary Material

1 SUPPLEMENTARY TABLES AND FIGURES

1.1 Tables

Table S1: Average numbers of case for 10 datasets of same percentages.

	10%	20%	30%	40%	50%	60%	70%	80%	90%	100%
Asthmastatus	2772	5556	8336	11108	13852	16613	19396	22163	24941	27692
COPDstatus	656	1309	1944	2581	3227	3864	4515	5154	5799	6449
Cancerstatus	119	242	363	485	600	720	838	957	1084	1202

Table S2: Number of patients who have asthma condition in each dataset.

	Data1	Data2	Data3	Data4	Data5	Data6	Data7	Data8	Data9	Data10
10%	2765	2788	2654	2736	2781	2781	2757	2744	2866	2848
20%	5499	5611	5429	5515	5576	5576	5638	5539	5612	5567
30%	8302	8427	8171	8304	8445	8445	8353	8316	8301	8295
40%	11112	11203	10909	11129	11191	11191	11085	11054	11137	11064
50%	13917	13963	13699	13880	13892	13892	13907	13759	13872	13742
60%	16622	16769	16465	16597	16601	16601	16698	16561	16616	16598
70%	19381	19509	19253	19344	19414	19414	19411	19394	19494	19344
80%	22160	22240	22072	22143	22166	22166	22219	22121	22203	22136
90%	24944	24965	24912	24934	24946	24946	24961	24943	24901	24961
100%	27692	27692	27692	27692	27692	27692	27692	27692	27692	27692

Table S3: Number of patients who have COPD condition in each dataset.

	Data1	Data2	Data3	Data4	Data5	Data6	Data7	Data8	Data9	Data10
10%	681	617	650	663	634	646	680	643	686	659
20%	1330	1249	1305	1326	1305	1337	1306	1285	1321	1321
30%	1957	1942	1953	1972	1955	1978	1905	1891	1929	1960
40%	2569	2608	2583	2626	2591	2623	2532	2554	2570	2560
50%	3212	3259	3222	3267	3225	3284	3237	3202	3201	3164
60%	3845	3861	3884	3897	3872	3878	3896	3846	3829	3832
70%	4505	4472	4520	4604	4513	4499	4555	4527	4483	4470
80%	5159	5159	5171	5208	5156	5133	5174	5134	5136	5107
90%	5816	5789	5827	5807	5805	5784	5800	5809	5766	5785
100%	6449	6449	6449	6449	6449	6449	6449	6449	6449	6449

Table S4: Number of patients who have lung cancer condition in each dataset.

	Data1	Data2	Data3	Data4	Data5	Data6	Data7	Data8	Data9	Data10
10%	128	122	109	122	125	117	113	109	123	117
20%	259	231	233	230	262	244	240	233	240	244
30%	372	373	349	368	384	363	353	353	350	364
40%	498	483	479	500	508	483	484	465	466	488
50%	622	602	600	598	614	612	593	582	572	604
60%	730	739	711	729	729	724	701	702	703	736
70%	830	849	837	843	856	826	837	814	821	865
80%	969	964	951	958	968	948	968	940	936	969
90%	1089	1079	1073	1090	1093	1094	1086	1082	1070	1080
100%	1202	1202	1202	1202	1202	1202	1202	1202	1202	1202

Table S5: Model evaluations of five prediction models on training 10%-100% datasets for predicting Asthma.

Dataset	Model	Precision	Recall	F1_Score	AUC
10%	ElasticNet	0.2404±0.0127	0.4535±0.0307	0.3135±0.0098	0.6566±0.0118
	XGBoost	0.2343±0.0163	0.4505±0.0360	0.3097±0.0123	0.6543±0.0095
	SVM	0.1701±0.0092	0.4800±0.0126	0.2635±0.0129	0.5655±0.0111
	LSTM	0.2036±0.0125	0.1780±0.0234	0.1891±0.0142	0.5787±0.0099
	DNN	0.1907±0.0143	0.4635±0.0432	0.2828±0.0121	0.5886±0.0078
20%	ElasticNet	0.2433±0.0087	0.4710±0.0238	0.3187±0.0071	0.6679±0.0070
	XGBoost	0.2412±0.0057	0.4501±0.0286	0.3137±0.0086	0.6638±0.0083
	SVM	0.1903±0.0057	0.4847±0.0131	0.2794±0.0036	0.5859±0.0063
	LSTM	0.2323±0.021	0.2787± 0.0232	0.2587±0.0118	0.6293±0.0084
	DNN	0.2235±0.0033	0.4654±0.0166	0.3034±0.0063	0.6063±0.0069
30%	ElasticNet	0.2416±0.0078	0.4729±0.0197	0.3195±0.0046	0.6705±0.0053
	XGBoost	0.2424±0.0057	0.4537±0.0213	0.3155±0.0055	0.6637±0.0060
	SVM	0.2004±0.0047	0.4888±0.0132	0.2886±0.0054	0.5956±0.0065
	LSTM	0.2373±0.0090	0.3553±0.0259	0.2840±0.0096	0.6398±0.0061
	DNN	0.2235±0.0049	0.4706±0.0315	0.3072±0.0069	0.6112±0.0074
40%	ElasticNet	0.2445±0.0091	0.4687±0.0270	0.3208±0.0047	0.6734±0.0038
	XGBoost	0.2448±0.0093	0.4550±0.0301	0.3177±0.0061	0.6707±0.0052
	SVM	0.2074±0.0066	0.4807±0.0180	0.2944±0.0057	0.6009±0.0043
	LSTM	0.2424±0.0096	0.3931±0.0305	0.2992±0.0095	0.6520±0.0063
	DNN	0.2239±0.0087	0.4665±0.0412	0.3106±0.0055	0.6120±0.0052
50%	ElasticNet	0.2410±0.0070	0.4836±0.0189	0.3214±0.0047	0.6744±0.0030
	XGBoost	0.2419±0.0085	0.4755±0.0264	0.3201±0.0047	0.6726±0.0041
	SVM	0.2108±0.0049	0.4844±0.0112	0.2966±0.0043	0.6027±0.0037
	LSTM	0.2382±0.0029	0.4398±0.0227	0.3088±0.0060	0.6593±0.0053

Table S5 continued from previous page

	DNN	0.2241±0.0062	0.4803±0.0286	0.3098±0.0032	0.6122±0.0044
60%	ElasticNet	0.2416±0.0087	0.4801±0.0155	0.3211±0.0056	0.6743±0.0035
	XGBoost	0.2443±0.0084	0.4644±0.0214	0.3198±0.0051	0.6733±0.0042
	SVM	0.2175±0.0055	0.4850±0.0220	0.3006±0.0051	0.6058±0.0040
	LSTM	0.2415±0.0090	0.4356±0.0309	0.3101±0.0066	0.6608±0.0049
	DNN	0.2305±0.0072	0.4781±0.0299	0.3117±0.0048	0.6142±0.0047
70%	ElasticNet	0.2436±0.0082	0.4751±0.0175	0.3217±0.0040	0.6743±0.0024
	XGBoost	0.2448±0.0049	0.4638±0.0148	0.3203±0.0015	0.6735±0.0031
	SVM	0.2181±0.0062	0.4851±0.0085	0.3028±0.0042	0.6080±0.0023
	LSTM	0.2367±0.0094	0.4575±0.0217	0.3115±0.0057	0.6637±0.0041
	DNN	0.2292±0.0104	0.4815±0.0353	0.3095±0.0053	0.6124±0.0044
80%	ElasticNet	0.2453±0.0068	0.4710±0.0214	0.3222±0.0029	0.6750±0.0032
	XGBoost	0.2466±0.0058	0.4595±0.0182	0.3207±0.0030	0.6741±0.0027
	SVM	0.2229±0.0059	0.4856±0.0177	0.3048±0.0034	0.6089±0.0028
	LSTM	0.2430±0.0051	0.4416±0.0184	0.3133±0.0030	0.6641±0.0033
	DNN	0.2345±0.0120	0.4638±0.0347	0.3105±0.0051	0.6119±0.0036
90%	ElasticNet	0.2459±0.0078	0.4671±0.0226	0.3218±0.0031	0.6745±0.0032
	XGBoost	0.2438±0.0045	0.4686±0.0102	0.3206±0.0026	0.6742±0.0024
	SVM	0.2229±0.0048	0.4880±0.0129	0.3061±0.0033	0.6103±0.0027
	LSTM	0.2377±0.0031	0.4457±0.0278	0.3098±0.0063	0.6595±0.0051
	DNN	0.2364±0.0045	0.4605±0.0168	0.3118±0.0047	0.6128±0.0041
100%	ElasticNet	0.2444±0.0055	0.4657±0.0148	0.3204±0.0019	0.6739±0.0030
	XGBoost	0.2446±0.0044	0.4669±0.0141	0.3209±0.0029	0.6737±0.0025
	SVM	0.2257±0.0032	0.4904±0.0094	0.3066±0.0031	0.6107±0.0031
	LSTM	0.2364±0.0073	0.4559±0.0243	0.3112±0.0032	0.6636±0.0026
	DNN	0.2380±0.0096	0.4542±0.0276	0.3117±0.0045	0.6128±0.0034

Table S6: Model evaluations of five prediction models on training 10%-100% datasets for predicting COPD.

Dataset	Model	Precision	Recall	F1_Score	AUC
10%	ElasticNet	0.2938±0.0415	0.3446±0.0524	0.3153±0.0386	0.8402±0.0154
	XGBoost	0.2863±0.0360	0.3447±0.0521	0.3053±0.0357	0.8261±0.0168
	SVM	0.1533±0.0180	0.2389±0.0464	0.1852±0.0227	0.5970±0.0198
	LSTM	0.1210±0.0886	0.0550±0.0443	0.0749±0.0191	0.6558±0.0176
	DNN	0.2149±0.0291	0.3055±0.0421	0.2451±0.0129	0.6334±0.0150
20%	ElasticNet	0.3115±0.0264	0.3758±0.0286	0.3399±0.0222	0.8473±0.0119
	XGBoost	0.3067±0.0249	0.3679±0.0270	0.3338±0.0212	0.8378±0.0100
	SVM	0.1997±0.0160	0.3028±0.0448	0.2394±0.0198	0.6315±0.0199
	LSTM	0.2871±0.0274	0.2208±0.0330	0.2482±0.0268	0.8084±0.0146
	DNN	0.2402±0.0079	0.3169±0.0369	0.2885±0.0186	0.6495±0.0167
30%	ElasticNet	0.3286±0.0226	0.3699±0.0312	0.3472±0.0206	0.8509±0.0110
	XGBoost	0.3099±0.0208	0.3544±0.0263	0.3296±0.0148	0.8396±0.0106

Table S6 continued from previous page

	SVM	0.2490±0.0270	0.3206±0.0330	0.2793±0.0239	0.6444±0.0156
	LSTM	0.3153±0.0222	0.2753±0.0233	0.2935±0.0199	0.8249±0.0143
	DNN	0.2687±0.0265	0.3348±0.0258	0.2974±0.0227	0.6523±0.0123
40%	ElasticNet	0.3310±0.0136	0.3714±0.0264	0.3496±0.0155	0.8568±0.0093
	XGBoost	0.3166±0.0126	0.3460±0.0225	0.3301±0.0164	0.8437±0.0090
	SVM	0.2686±0.0177	0.3415±0.0290	0.2978±0.0138	0.6556±0.0132
	LSTM	0.3150±0.0223	0.3205±0.0269	0.3065±0.0202	0.8320±0.0088
	DNN	0.2753±0.0365	0.3360±0.0337	0.2999±0.0218	0.6533±0.0147
50%	ElasticNet	0.3339±0.0160	0.3845±0.0222	0.3571±0.0154	0.8603±0.0083
	XGBoost	0.3137±0.0161	0.3570±0.0204	0.3335±0.0137	0.8429±0.0073
	SVM	0.2847±0.0187	0.3457±0.0201	0.3120±0.0172	0.6590±0.0100
	LSTM	0.3237±0.0144	0.3213±0.0201	0.3171±0.0154	0.8417±0.0104
	DNN	0.2844±0.0268	0.3365±0.0371	0.3063±0.0191	0.6545±0.0166
60%	ElasticNet	0.3353±0.0140	0.3839±0.0204	0.3576±0.0120	0.8635±0.0077
	XGBoost	0.3105±0.0081	0.3607±0.0206	0.3335±0.0110	0.8444±0.0063
	SVM	0.2874±0.0188	0.3557±0.0212	0.3175±0.0164	0.6637±0.0102
	LSTM	0.3236±0.0144	0.3322±0.0212	0.3267±0.0154	0.8464±0.0104
	DNN	0.2830±0.0177	0.3307±0.0231	0.3148±0.0165	0.6639±0.0139
70%	ElasticNet	0.3455±0.0265	0.3802±0.0158	0.3612±0.0135	0.8646±0.0065
	XGBoost	0.3104±0.0087	0.3608±0.0249	0.3332±0.0109	0.8433±0.0057
	SVM	0.3033±0.0119	0.3546±0.0312	0.3261±0.0131	0.6643±0.0141
	LSTM	0.3235±0.0185	0.3330±0.0276	0.3272±0.0155	0.8453±0.0062
	DNN	0.2973±0.0041	0.3361±0.0248	0.3149±0.0122	0.6553±0.0114
80%	ElasticNet	0.3512±0.0205	0.3776±0.0137	0.3637±0.0146	0.8649±0.0068
	XGBoost	0.3184±0.0115	0.3530±0.0231	0.3344±0.0131	0.8442±0.0056
	SVM	0.3090±0.0133	0.3671±0.0298	0.3347±0.0129	0.6704±0.0135
	LSTM	0.3234±0.0217	0.3387±0.0281	0.3294±0.0135	0.8455±0.0071
	DNN	0.2918±0.0126	0.3416±0.0211	0.3121±0.0081	0.6553±0.0092
90%	ElasticNet	0.3498±0.0132	0.3864±0.0126	0.3671±0.0106	0.8658±0.0070
	XGBoost	0.3154±0.0180	0.3531±0.0180	0.3326±0.0109	0.8449±0.0059
	SVM	0.3162±0.0133	0.3567±0.0189	0.3349±0.0112	0.6660±0.0088
	LSTM	0.3203±0.0106	0.3387±0.0214	0.3287±0.0097	0.8446±0.0071
	DNN	0.2981±0.0237	0.3390±0.0265	0.3159±0.0150	0.6599±0.0118
100%	ElasticNet	0.3549±0.0112	0.3870±0.0205	0.3699±0.0110	0.8660±0.0071
	XGBoost	0.3136±0.0141	0.3501±0.0148	0.3307±0.0130	0.8429±0.0066
	SVM	0.3247±0.0135	0.3562±0.0206	0.3394±0.0125	0.6661±0.0097
	LSTM	0.3143±0.0172	0.3418±0.0223	0.3269±0.0145	0.8412±0.0092
	DNN	0.2907±0.0239	0.3359±0.0241	0.3106±0.0157	0.6546±0.0110

Table S7: Model evaluations of five prediction models on training 10%-100% datasets for predicting lung cancer.

Dataset	Model	Precision	Recall	F1_Score	AUC
10%	ElasticNet	0.0619±0.0282	0.1573±0.1306	0.0814±0.0357	0.8010±0.0460
	XGBoost	0.0319±0.0156	0.1490±0.1016	0.0490±0.0219	0.7134±0.0636
	SVM	0.0478±0.0272	0.1413±0.0985	0.0659±0.0310	0.5601±0.0420
	LSTM	0.0341±0.0250	0.0554±0.0003	0.0273±0.0030	0.5248±0.1088
	DNN	0.0398±0.0271	0.1036±0.1006	0.0402±0.0341	0.5513±0.0331
20%	ElasticNet	0.1259±0.0459	0.2161±0.0471	0.1557±0.0474	0.8612±0.0291
	XGBoost	0.0507±0.0178	0.1280±0.0599	0.0701±0.0228	0.7830±0.0201
	SVM	0.1119±0.0278	0.1405±0.0679	0.1192±0.0392	0.5669±0.0329
	LSTM	0.0419±0.0270	0.0511±0.0012	0.0449±0.0053	0.5392±0.0183
	DNN	0.0426±0.0339	0.1144±0.0484	0.0437±0.0354	0.5531±0.0198
30%	ElasticNet	0.1568±0.0357	0.1959±0.0413	0.1704±0.0264	0.8738±0.0209
	XGBoost	0.0547±0.0119	0.1394±0.0298	0.0765±0.0108	0.8027±0.0162
	SVM	0.1234±0.0295	0.1456±0.0365	0.1316±0.0295	0.5697±0.0179
	LSTM	0.1509±0.0340	0.0721±0.0322	0.0975±0.0354	0.8134±0.0195
	DNN	0.0463±0.0377	0.1387±0.0562	0.0430±0.0348	0.5791±0.0366
40%	ElasticNet	0.1701±0.0428	0.2097±0.0343	0.1855±0.0325	0.8812±0.0231
	XGBoost	0.0602±0.0140	0.1432±0.0354	0.0839±0.0183	0.8109±0.0191
	SVM	0.1345±0.0311	0.1376±0.0447	0.1329±0.0301	0.5662±0.0219
	LSTM	0.1605±0.0494	0.0823±0.0364	0.1062±0.0415	0.8314±0.0251
	DNN	0.0437±0.0227	0.1517±0.0415	0.0508±0.0296	0.5600±0.0131
50%	ElasticNet	0.2113±0.0353	0.2163±0.0320	0.2110±0.0235	0.8990±0.0149
	XGBoost	0.0616±0.0122	0.1702±0.0361	0.0885±0.0128	0.8168±0.0196
	SVM	0.1616±0.0238	0.1767±0.0397	0.1656±0.0239	0.5856±0.0193
	LSTM	0.1655±0.0396	0.0929±0.0301	0.1146±0.0269	0.8376±0.0211
	DNN	0.0476±0.0131	0.1597±0.0406	0.0547±0.0187	0.5779±0.0197
60%	ElasticNet	0.2197±0.0211	0.2504±0.0225	0.2327±0.0119	0.9090±0.0132
	XGBoost	0.0663±0.0104	0.1680±0.0373	0.0940±0.0127	0.8182±0.0179
	SVM	0.1758±0.0204	0.2027±0.0423	0.1854±0.0226	0.5985±0.0205
	LSTM	0.1754±0.0664	0.1139±0.0244	0.1338±0.0281	0.8480±0.0159
	DNN	0.0527±0.0198	0.1690±0.0577	0.0598±0.0262	0.5698±0.0260
70%	ElasticNet	0.2500±0.0362	0.2291±0.0292	0.2381±0.0278	0.9130±0.0109
	XGBoost	0.0662±0.0271	0.1908±0.0400	0.0933±0.0115	0.8188±0.0160
	SVM	0.1804±0.0180	0.2132±0.0417	0.1938±0.0230	0.6038±0.0204
	LSTM	0.1680±0.0449	0.1198±0.0414	0.1352±0.0352	0.8443±0.0133
	DNN	0.0464±0.0254	0.1702±0.0496	0.0635±0.0319	0.5613±0.0216
80%	ElasticNet	0.2546±0.0213	0.2579±0.0280	0.2551±0.0168	0.9181±0.0101
	XGBoost	0.0668±0.0068	0.1767±0.0290	0.0964±0.0092	0.8226±0.0142
	SVM	0.2001±0.0232	0.2271±0.0390	0.2104±0.0198	0.6108±0.0189
	LSTM	0.1606±0.0416	0.1261±0.0221	0.1393±0.0249	0.8395±0.0404
	DNN	0.0432±0.0176	0.1766±0.0440	0.0642±0.0199	0.5707±0.0242
90%	ElasticNet	0.2736±0.0220	0.2560±0.0353	0.2621±0.0165	0.9231±0.0089

Table S7 continued from previous page

	XGBoost	0.0624±0.0079	0.1965±0.0567	0.0933±0.0098	0.8193±0.0134
	SVM	0.2287±0.0200	0.2147±0.0298	0.2201±0.0178	0.6052±0.0146
	LSTM	0.1708±0.0472	0.1278±0.0267	0.1393±0.0208	0.8541±0.0149
	DNN	0.0549±0.0209	0.1802±0.0589	0.0751±0.0204	0.5673±0.0202
100%	ElasticNet	0.2783±0.0219	0.2704±0.0304	0.2731±0.0184	0.9247±0.0077
	XGBoost	0.0635±0.0063	0.1610±0.0392	0.0899±0.0101	0.8219±0.0135
	SVM	0.2310±0.0244	0.2358±0.0284	0.2313±0.0120	0.6155±0.0137
	LSTM	0.1833±0.0290	0.1279±0.0320	0.1483±0.0250	0.8563±0.0142
	DNN	0.0551±0.0218	0.1840±0.0383	0.0857±0.0264	0.5685±0.0133

1.2 Figures

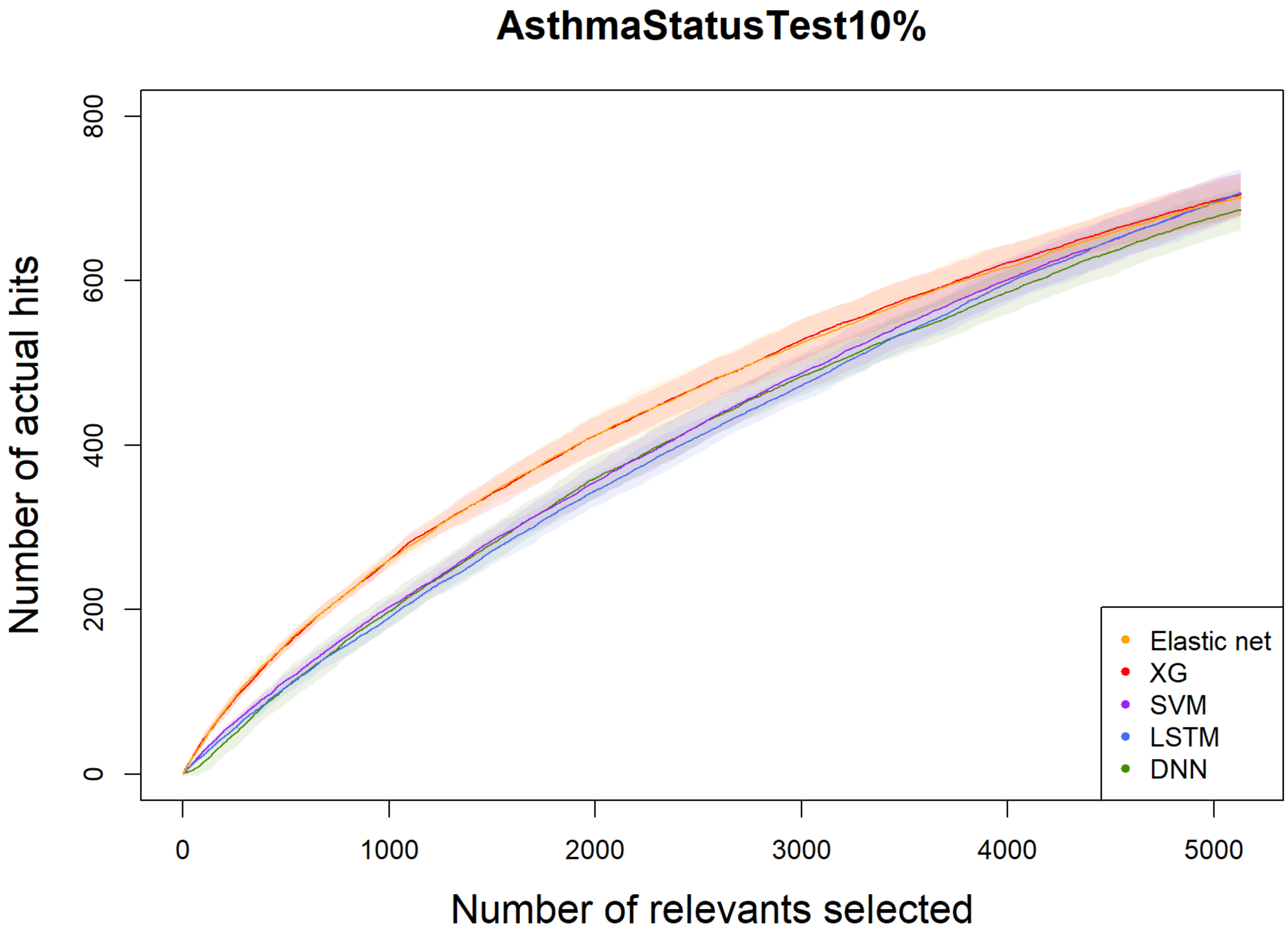

Figure S1. Hit curve on asthma for 10% dataset.

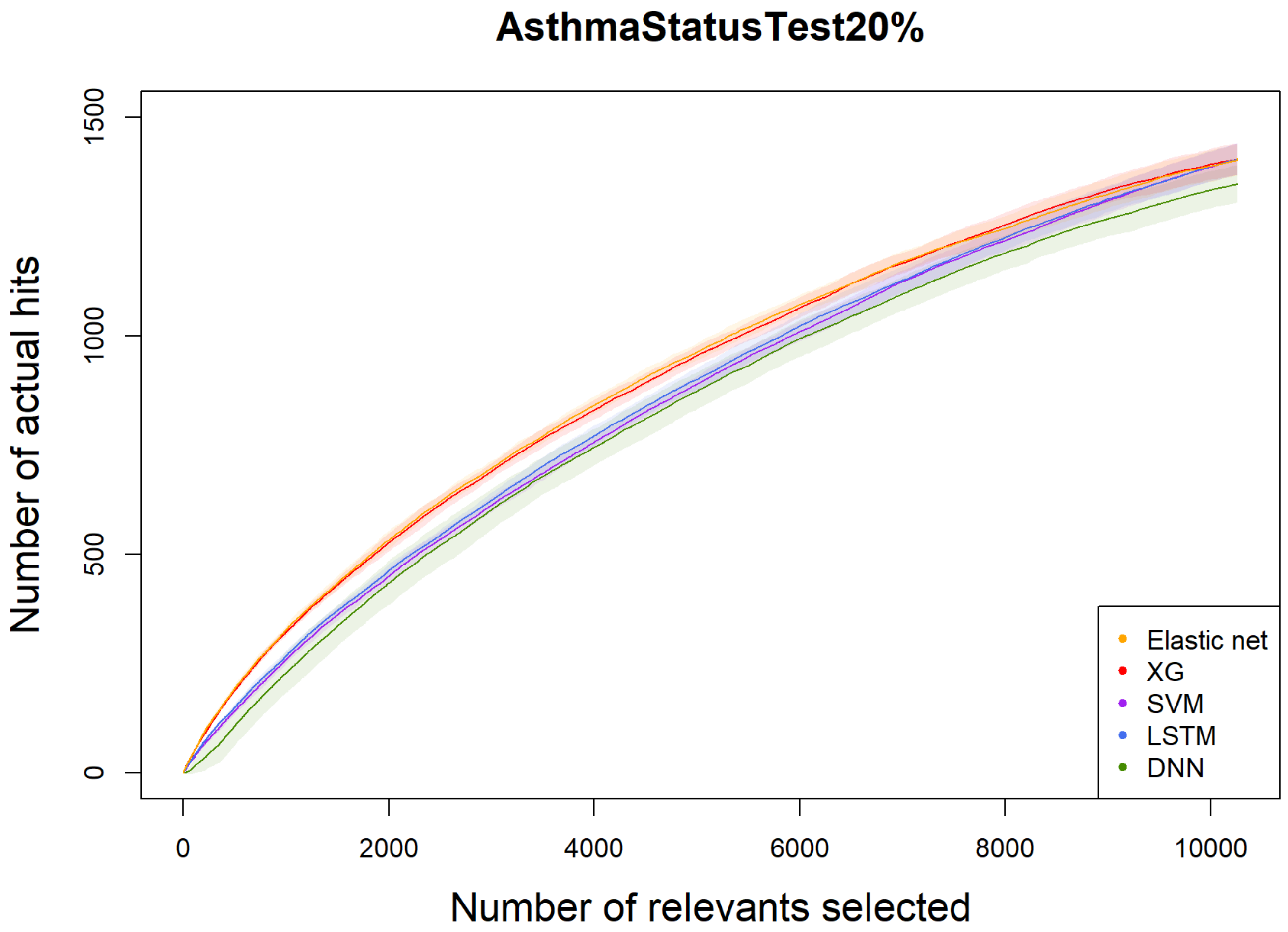

Figure S2. Hit curve on asthma for 20% dataset.

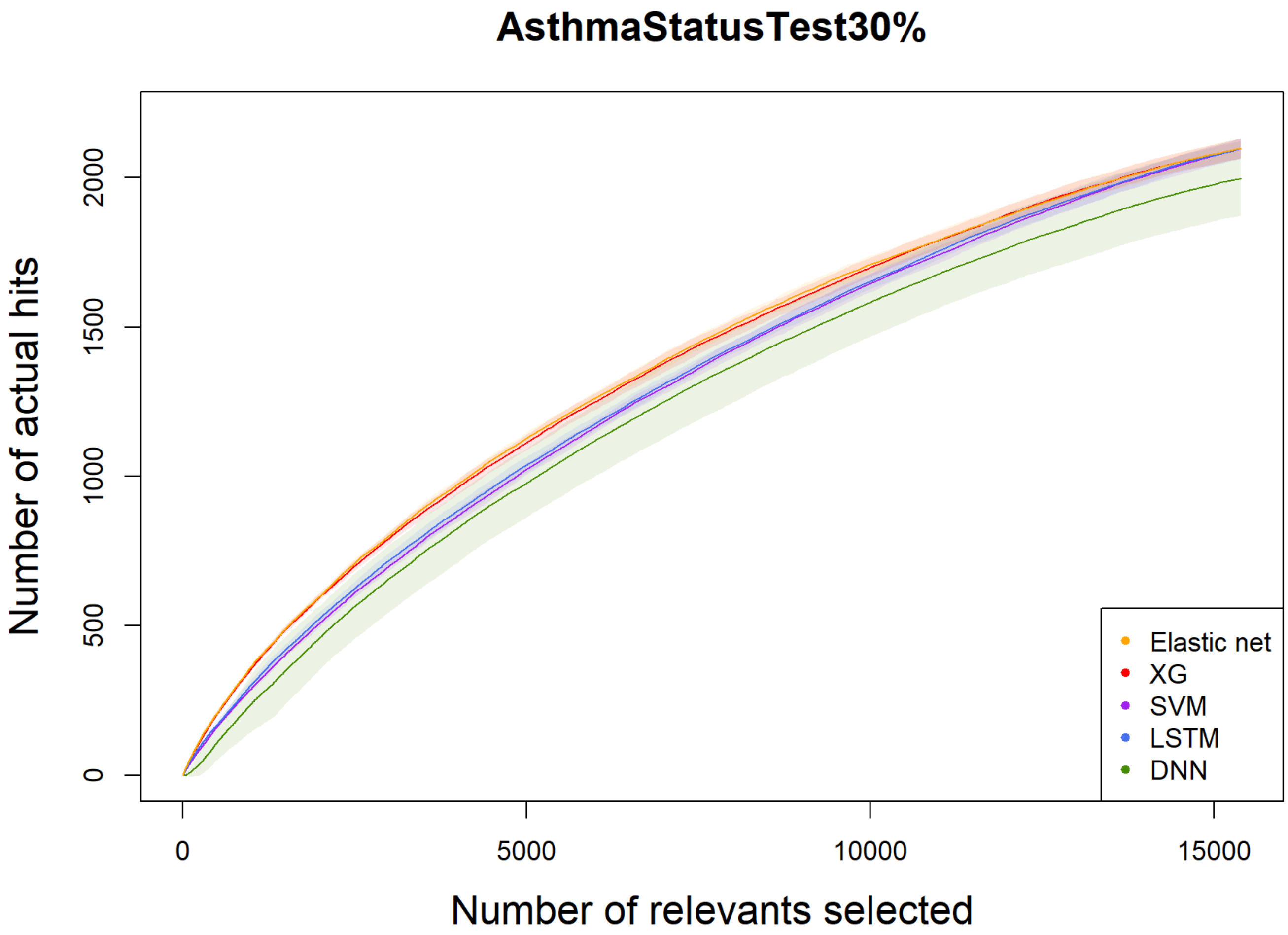

Figure S3. Hit curve on asthma for 30% dataset.

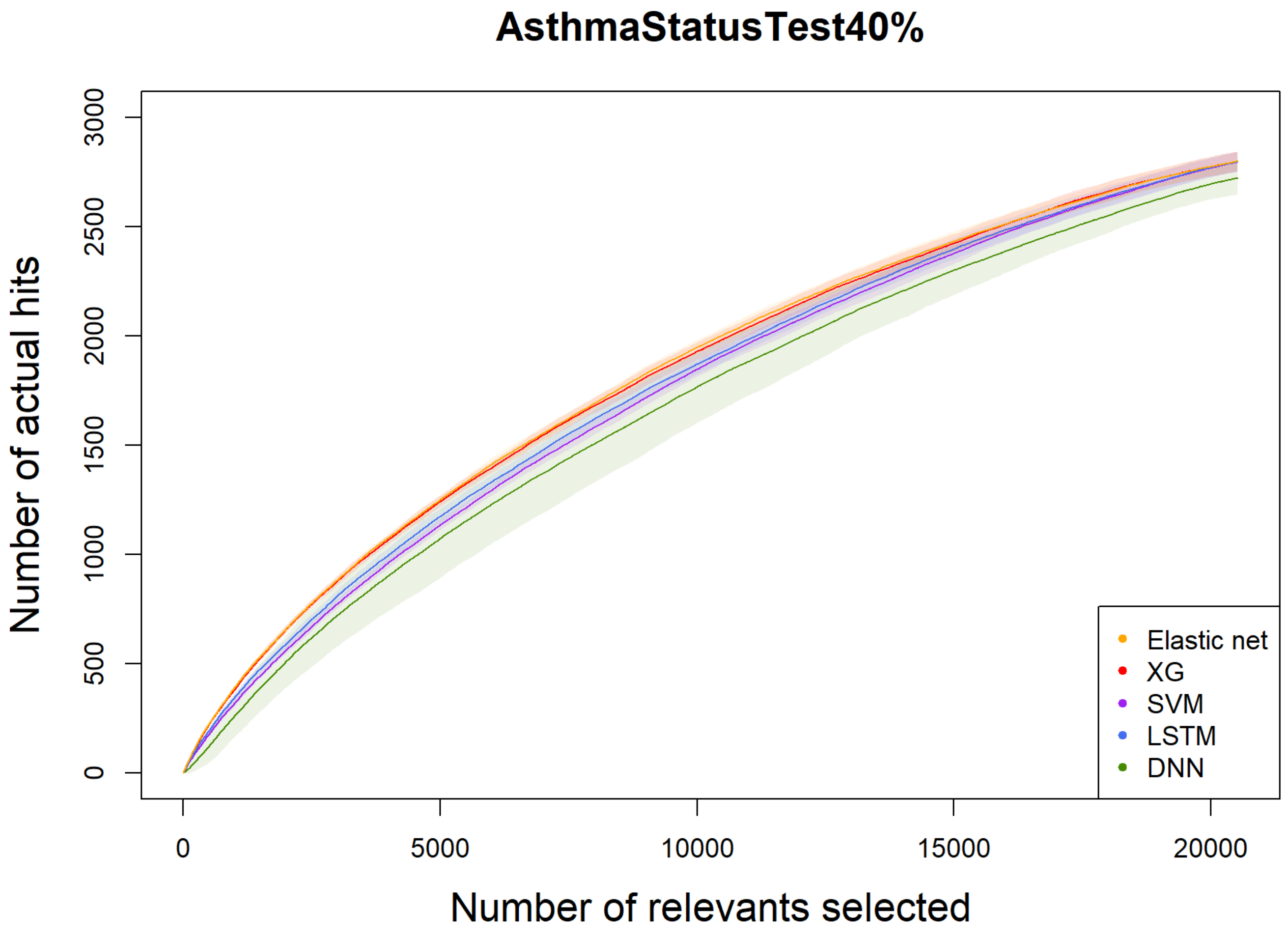

Figure S4. Hit curve on asthma for 40% dataset.

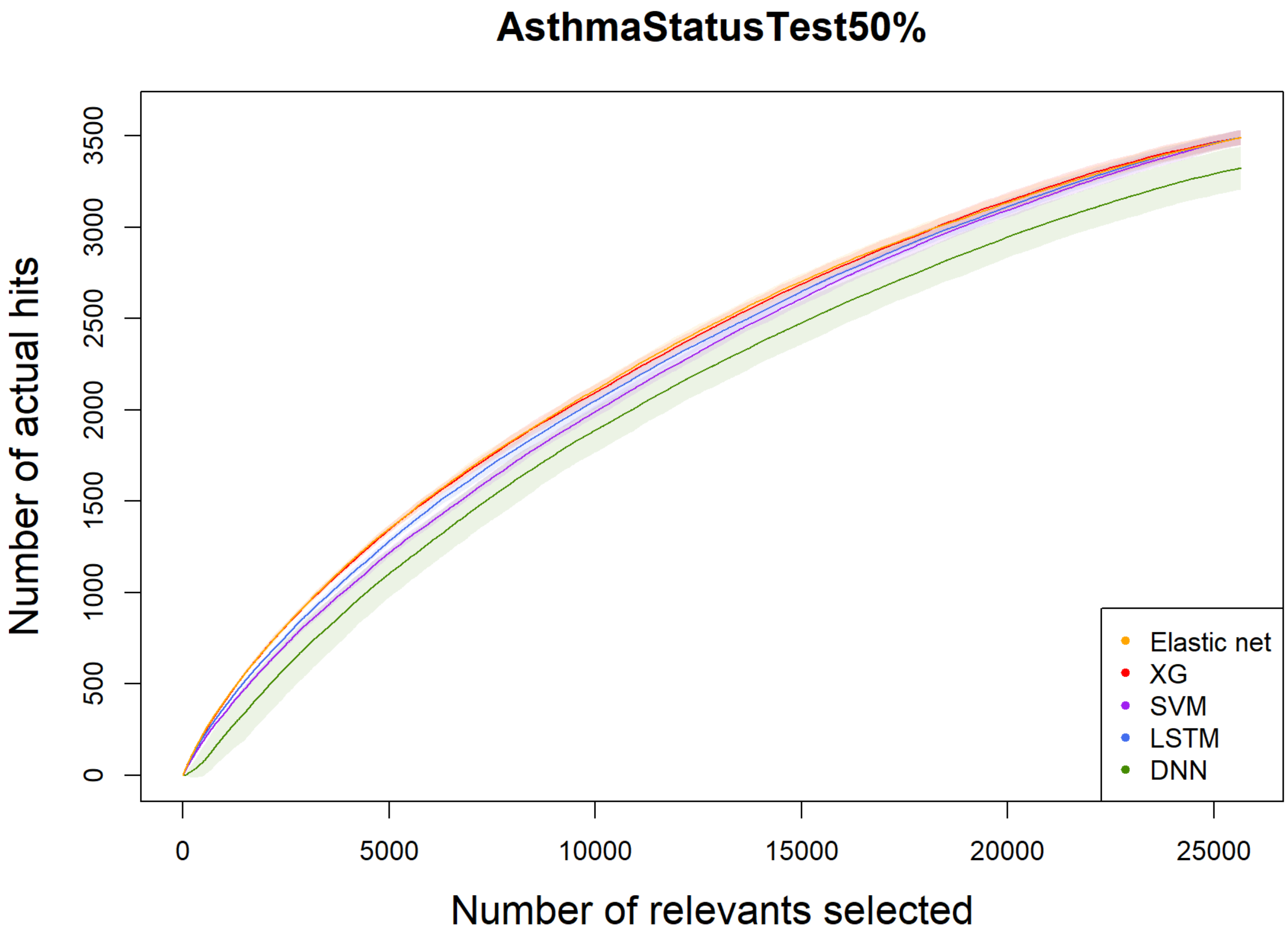

Figure S5. Hit curve on asthma for 50% dataset.

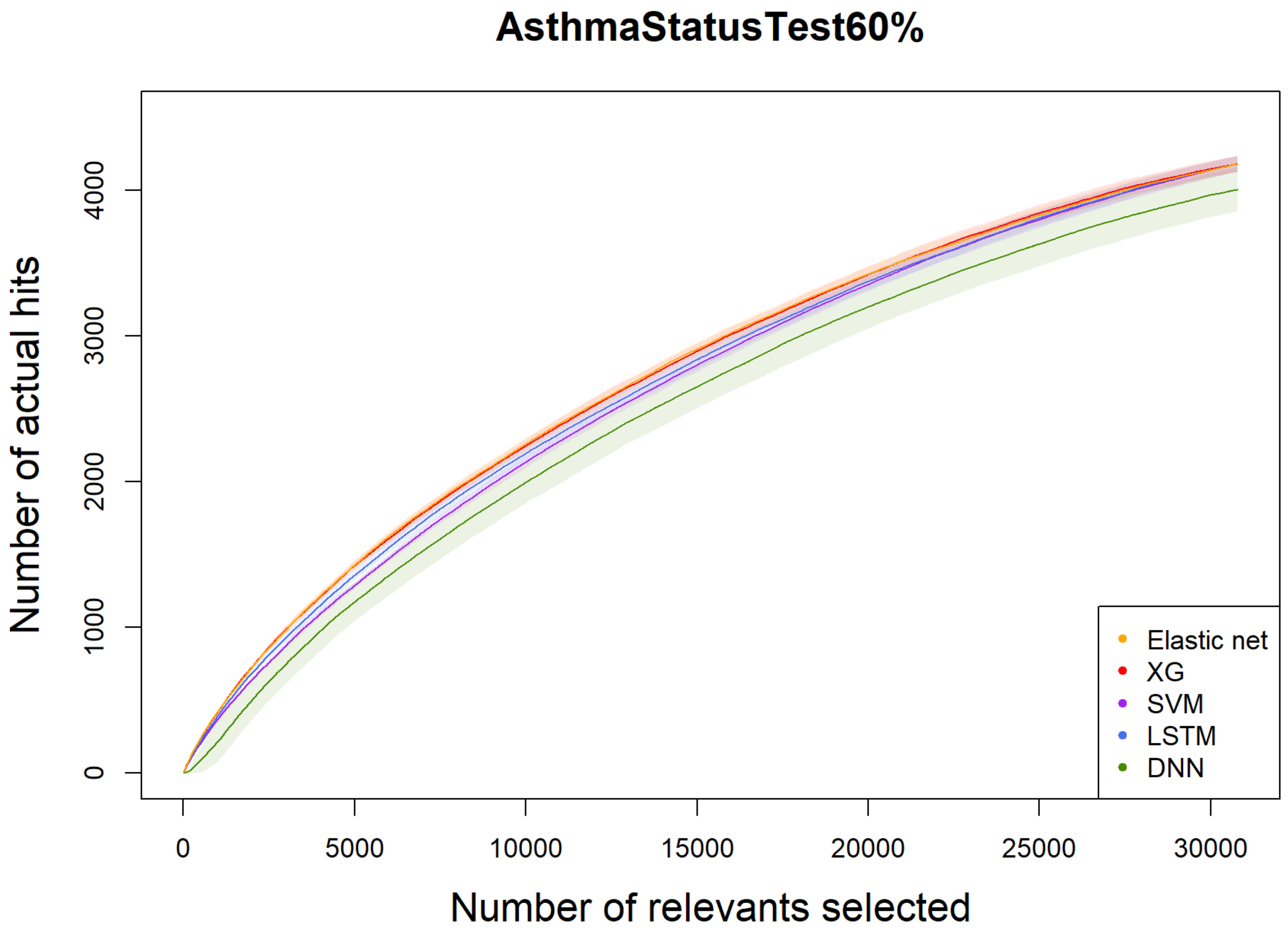

Figure S6. Hit curve on asthma for 60% dataset.

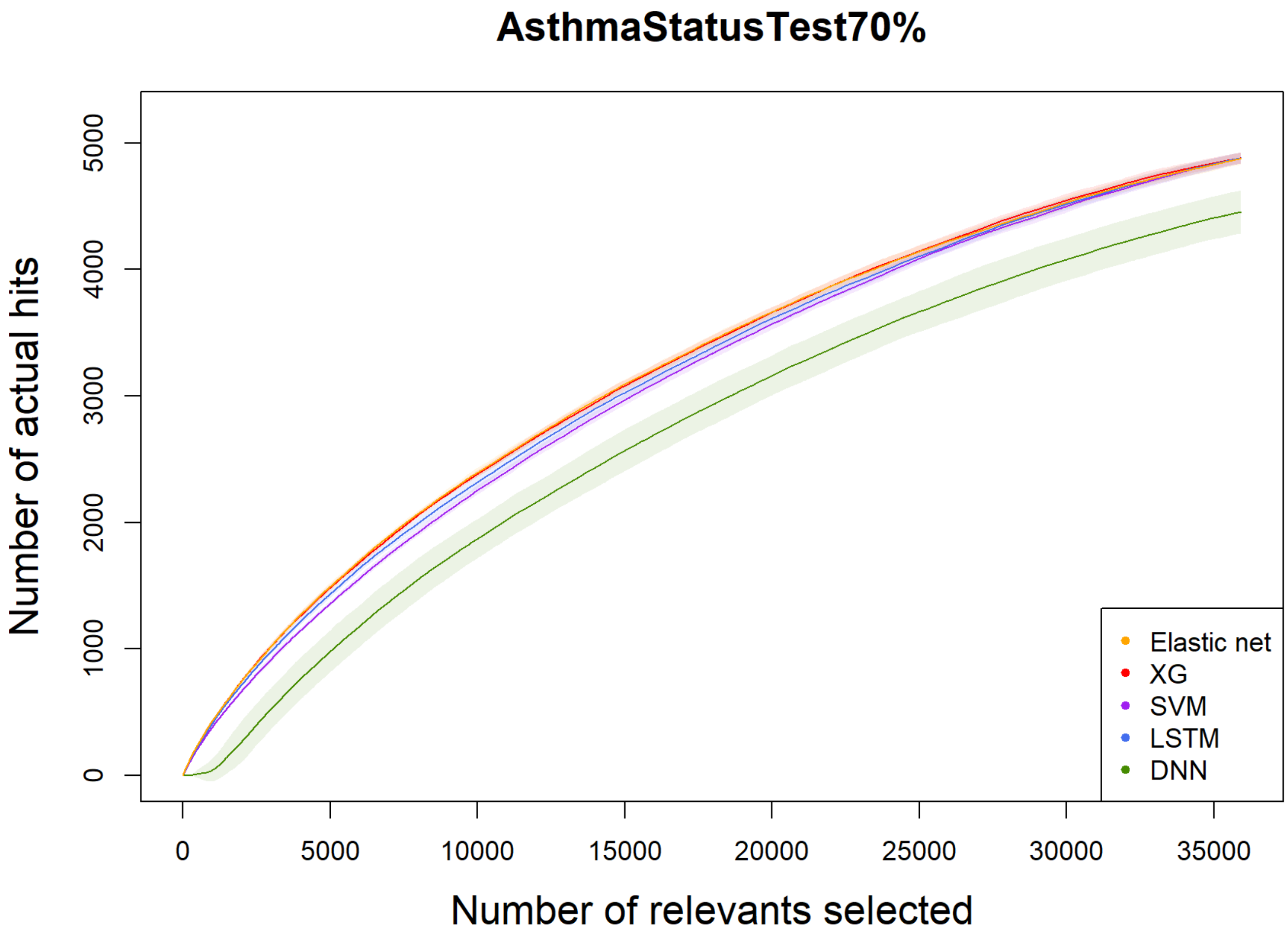

Figure S7. Hit curve on asthma for 70% dataset.

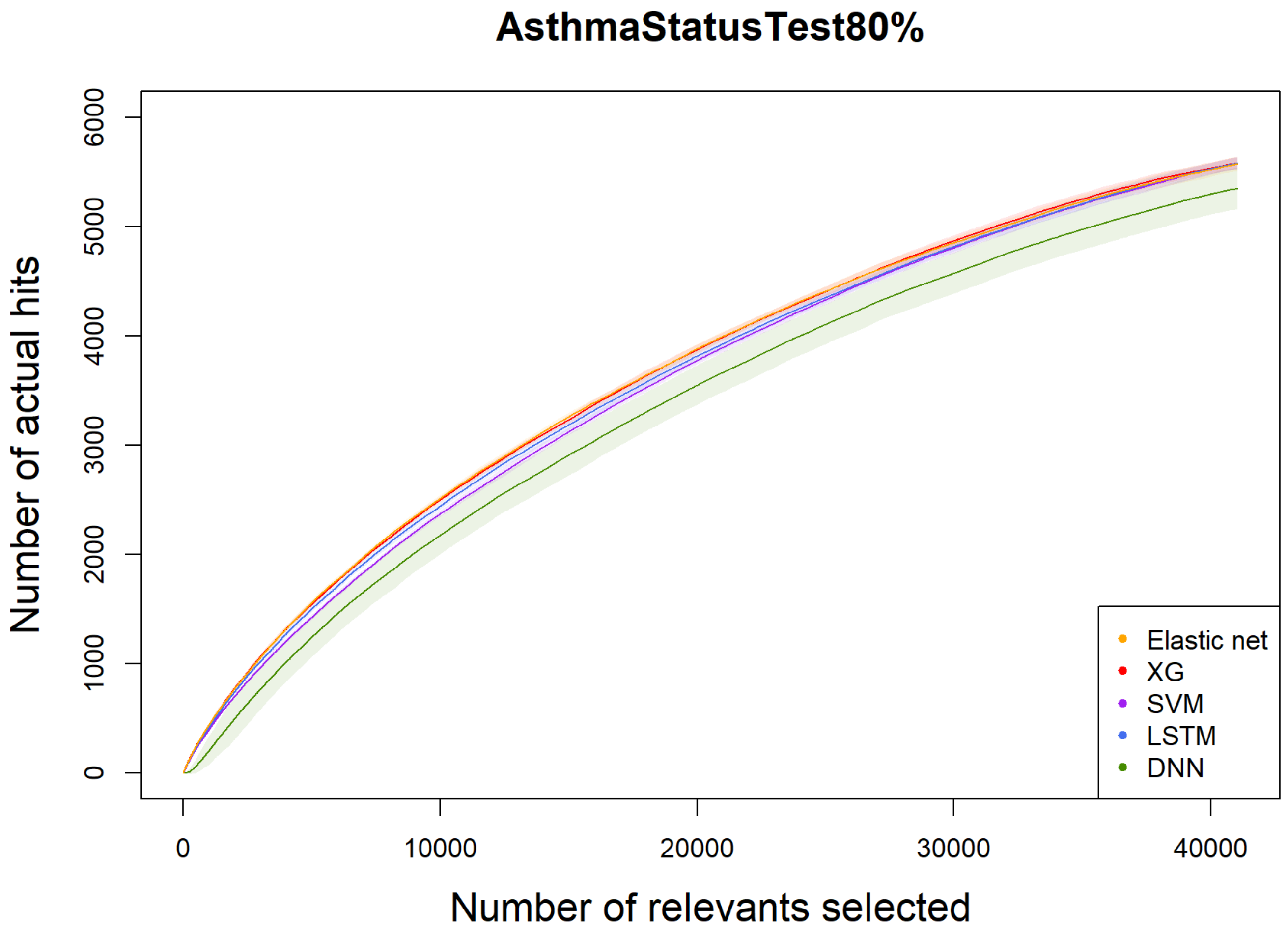

Figure S8. Hit curve on asthma for 80% dataset.

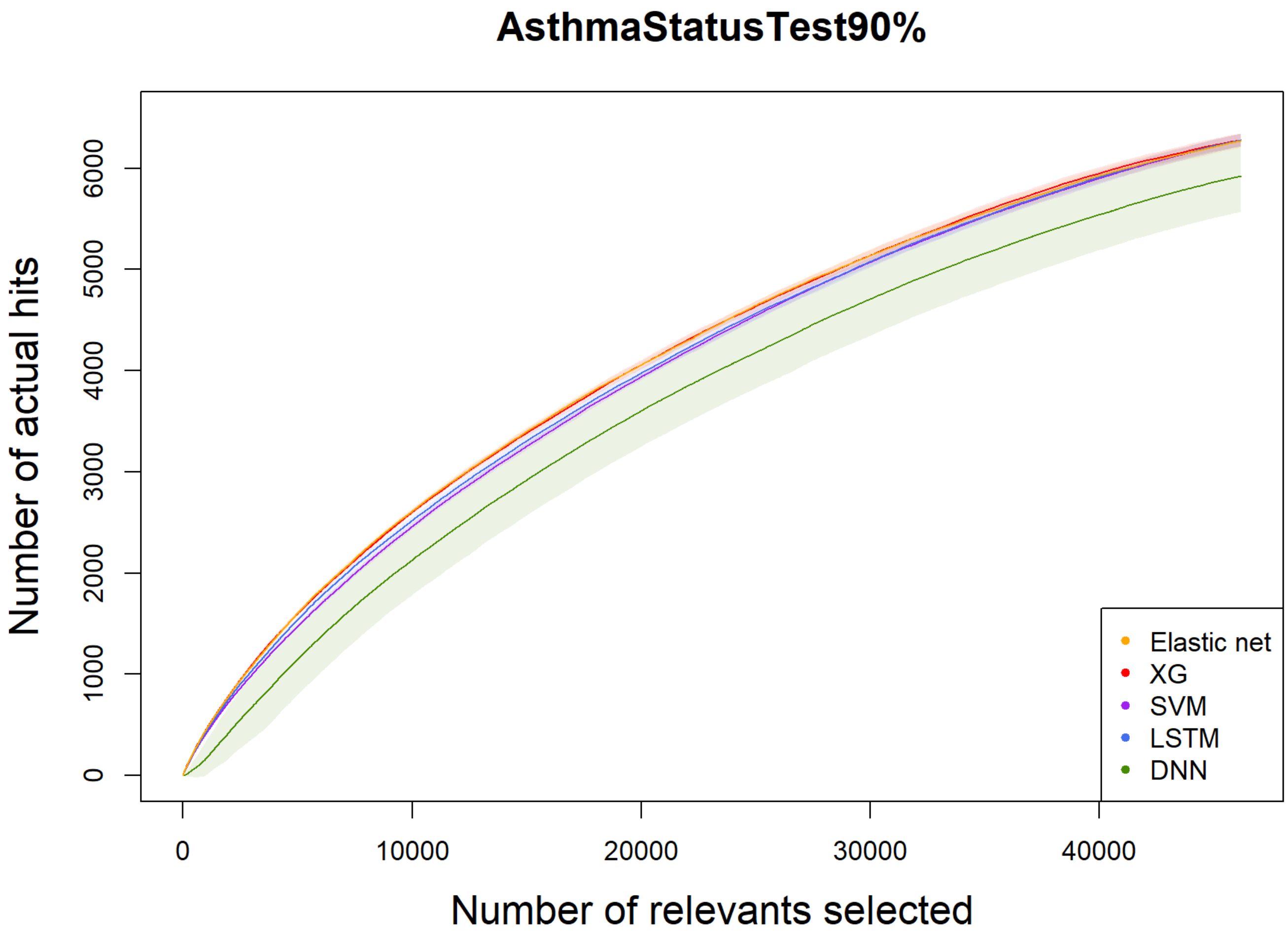

Figure S9. Hit curve on asthma for 90% dataset.

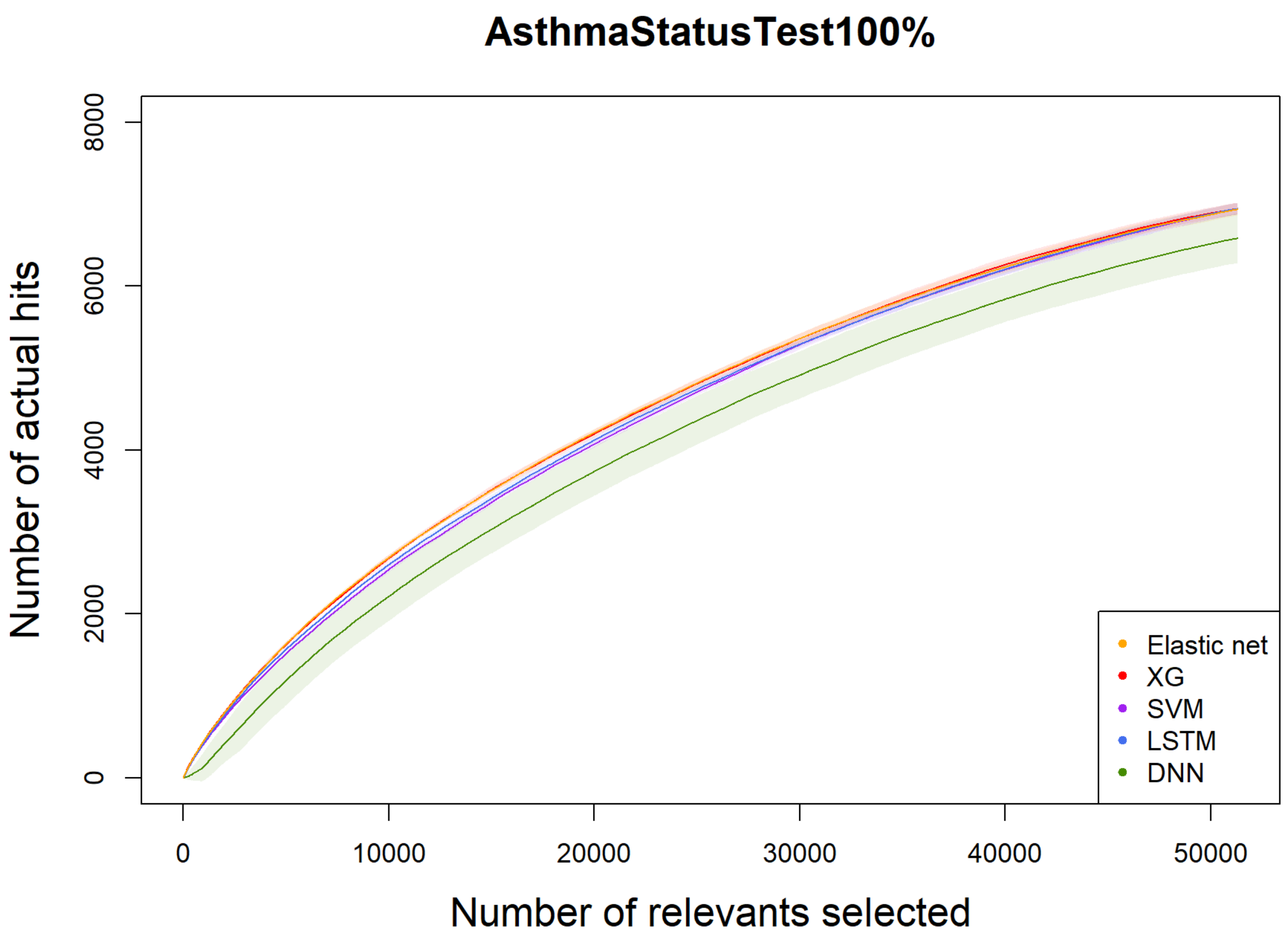

Figure S10. Hit curve on asthma for 100% dataset.

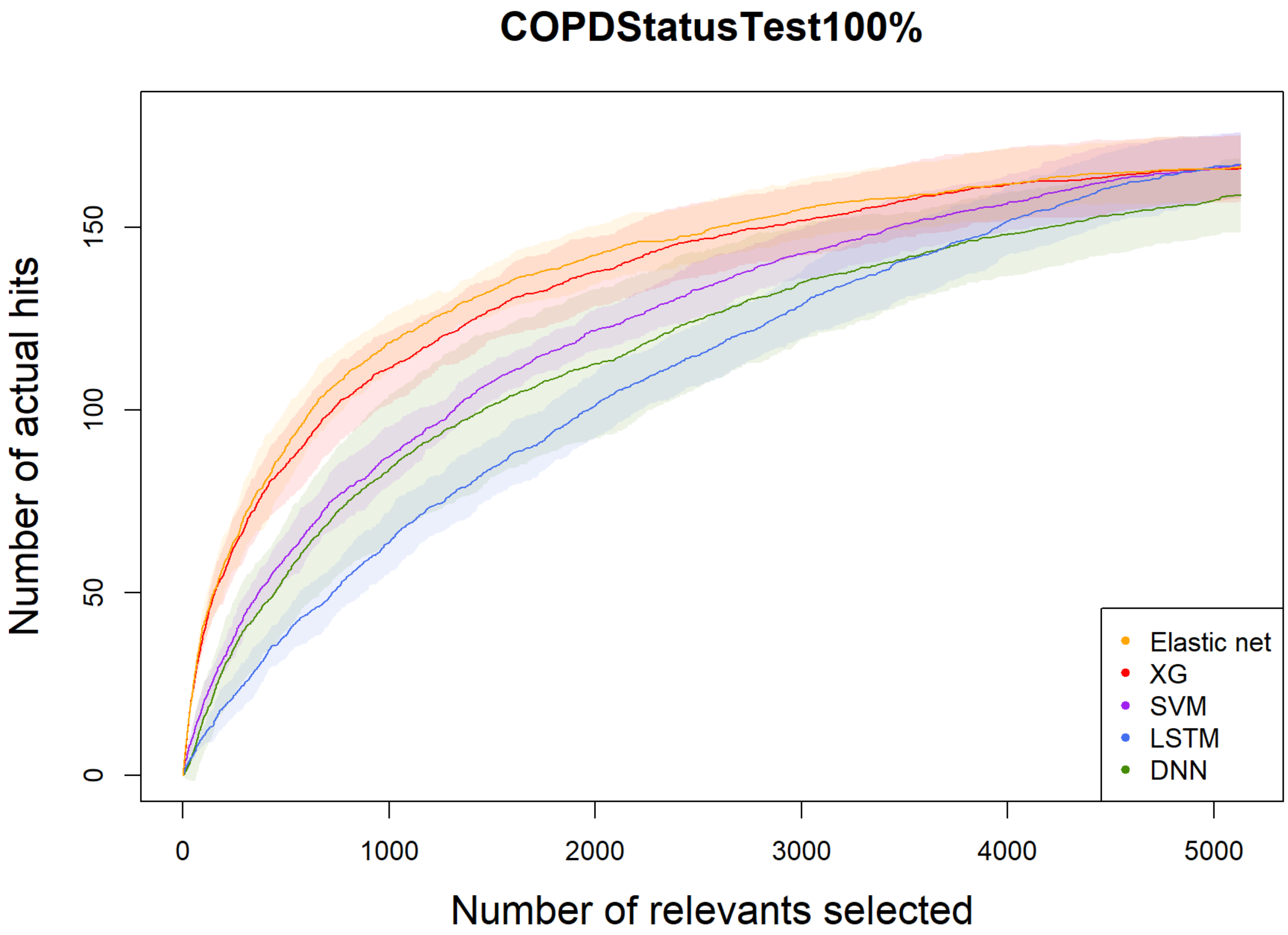

Figure S11. Hit curve on COPD for 10% dataset.

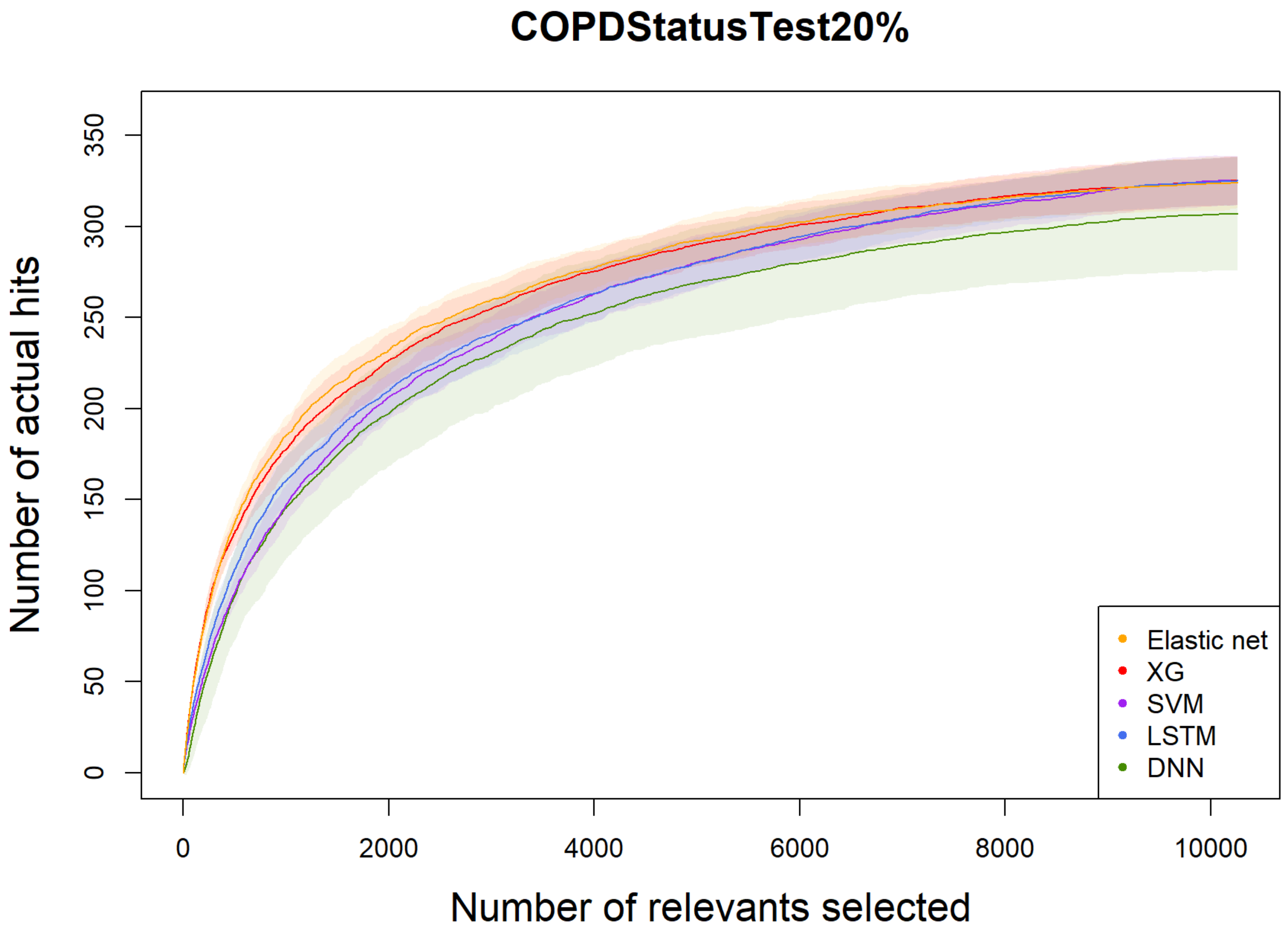

Figure S12. Hit curve on COPD for 20% dataset.

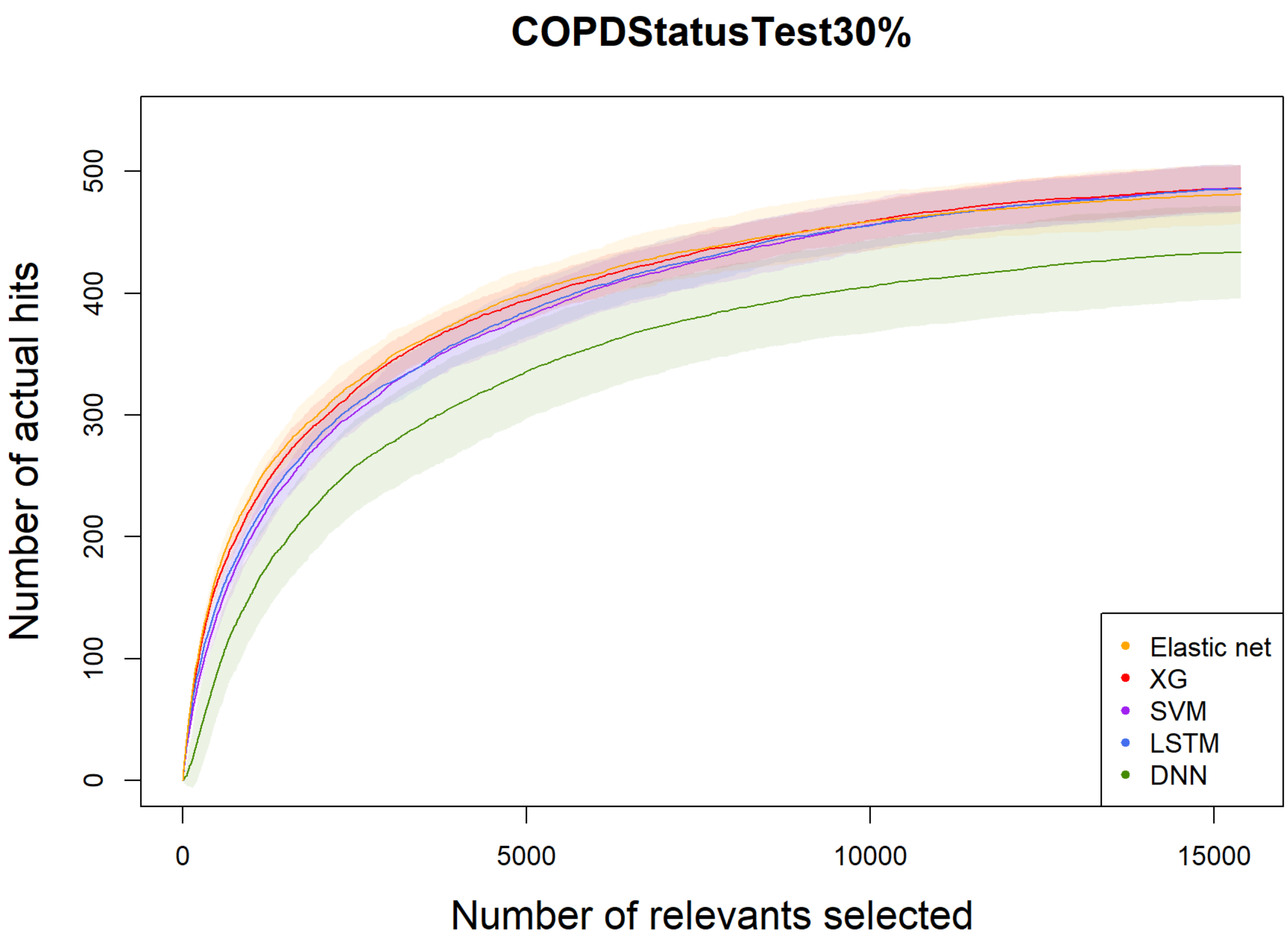

Figure S13. Hit curve on COPD for 30% dataset.

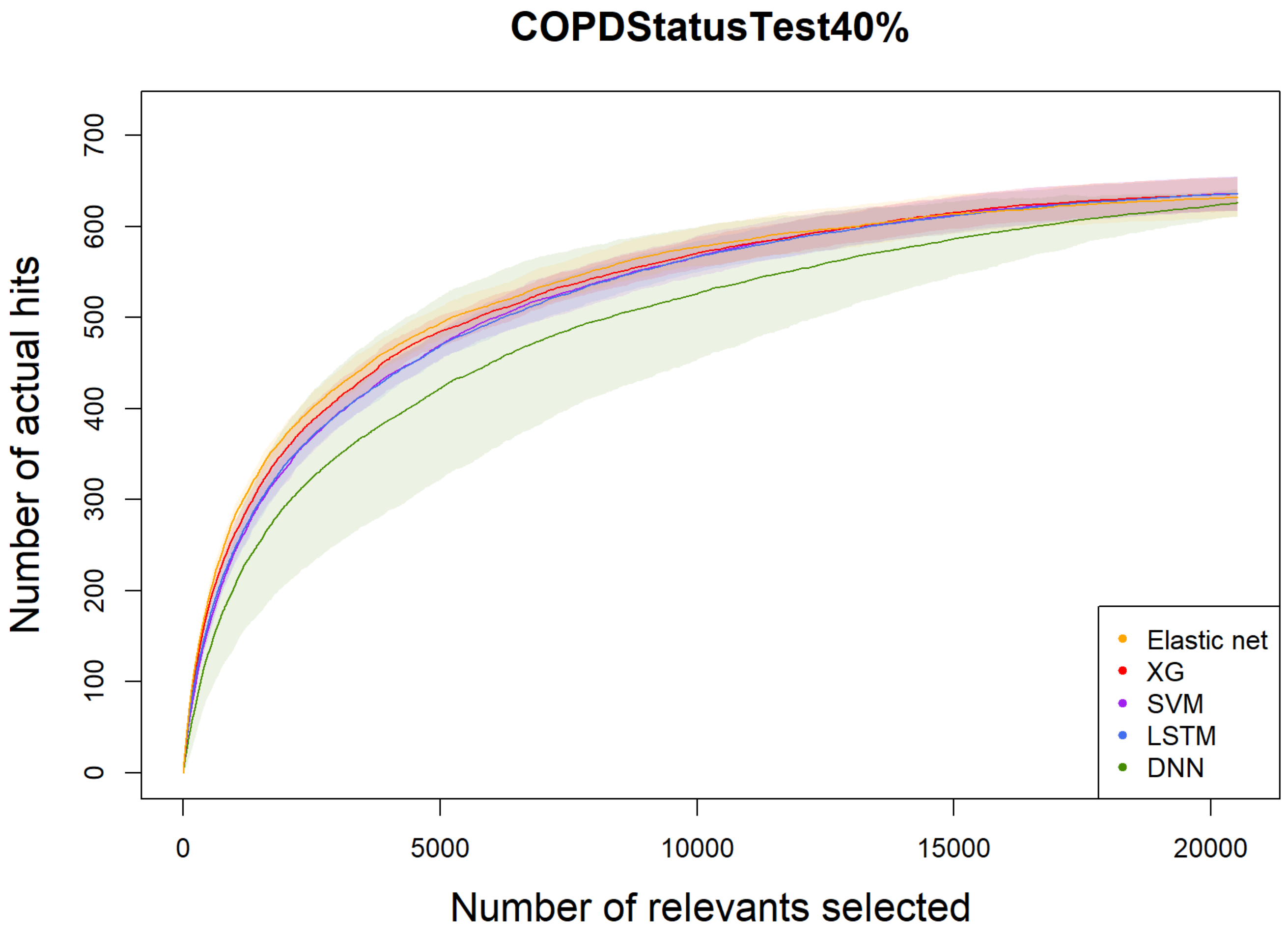

Figure S14. Hit curve on COPD for 40% dataset.

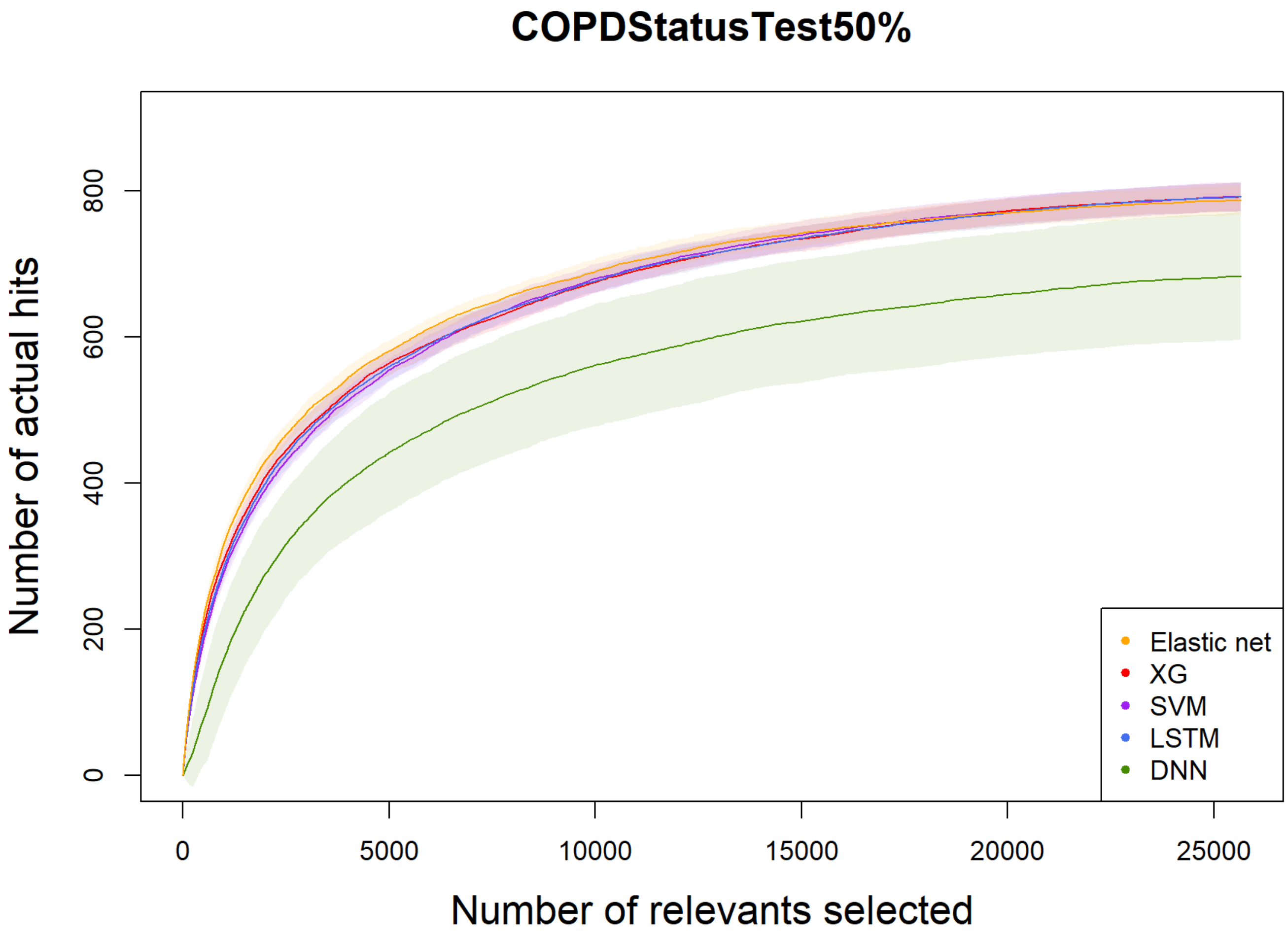

Figure S15. Hit curve on COPD for 50% dataset.

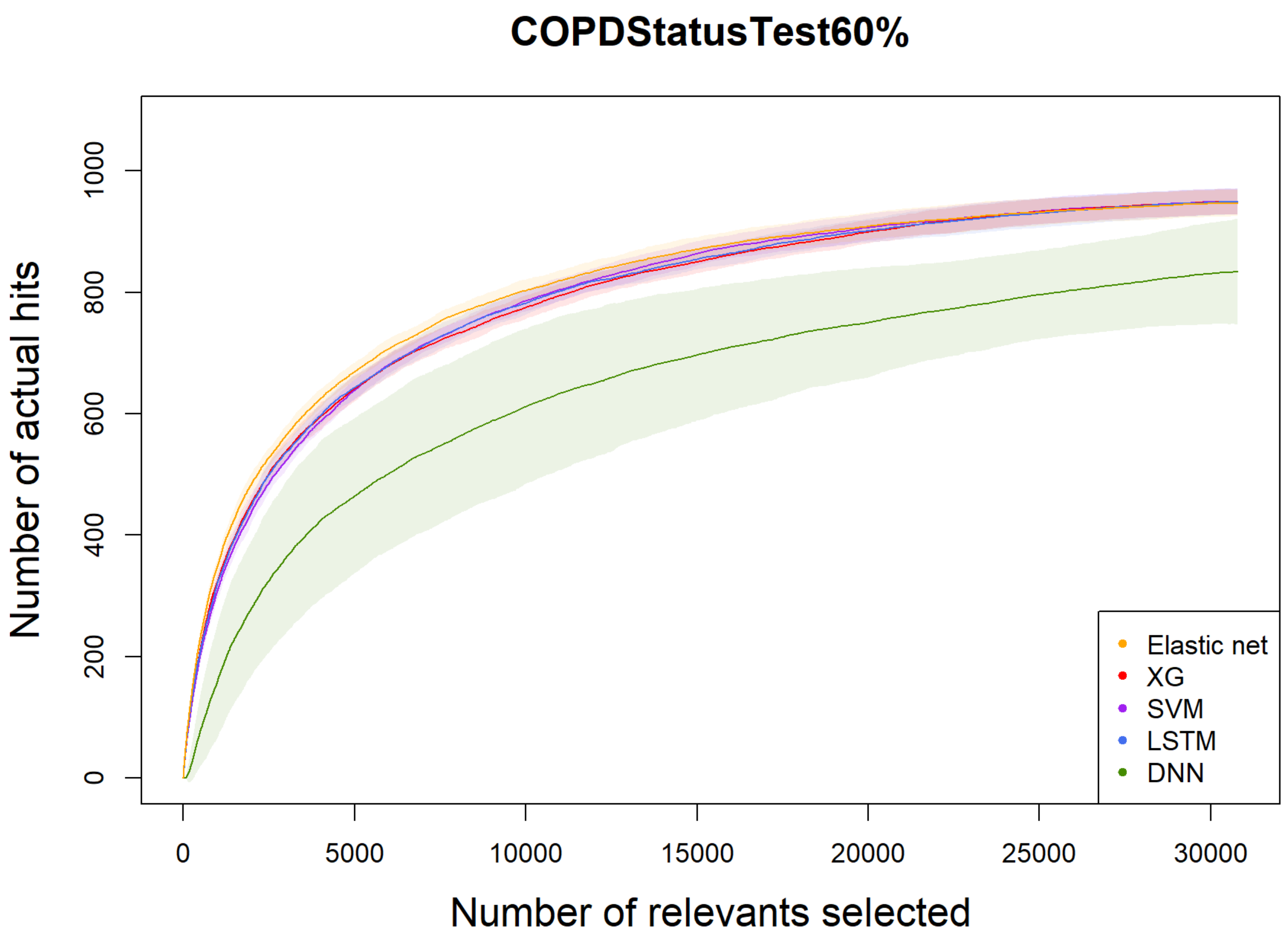

Figure S16. Hit curve on COPD for 60% dataset.

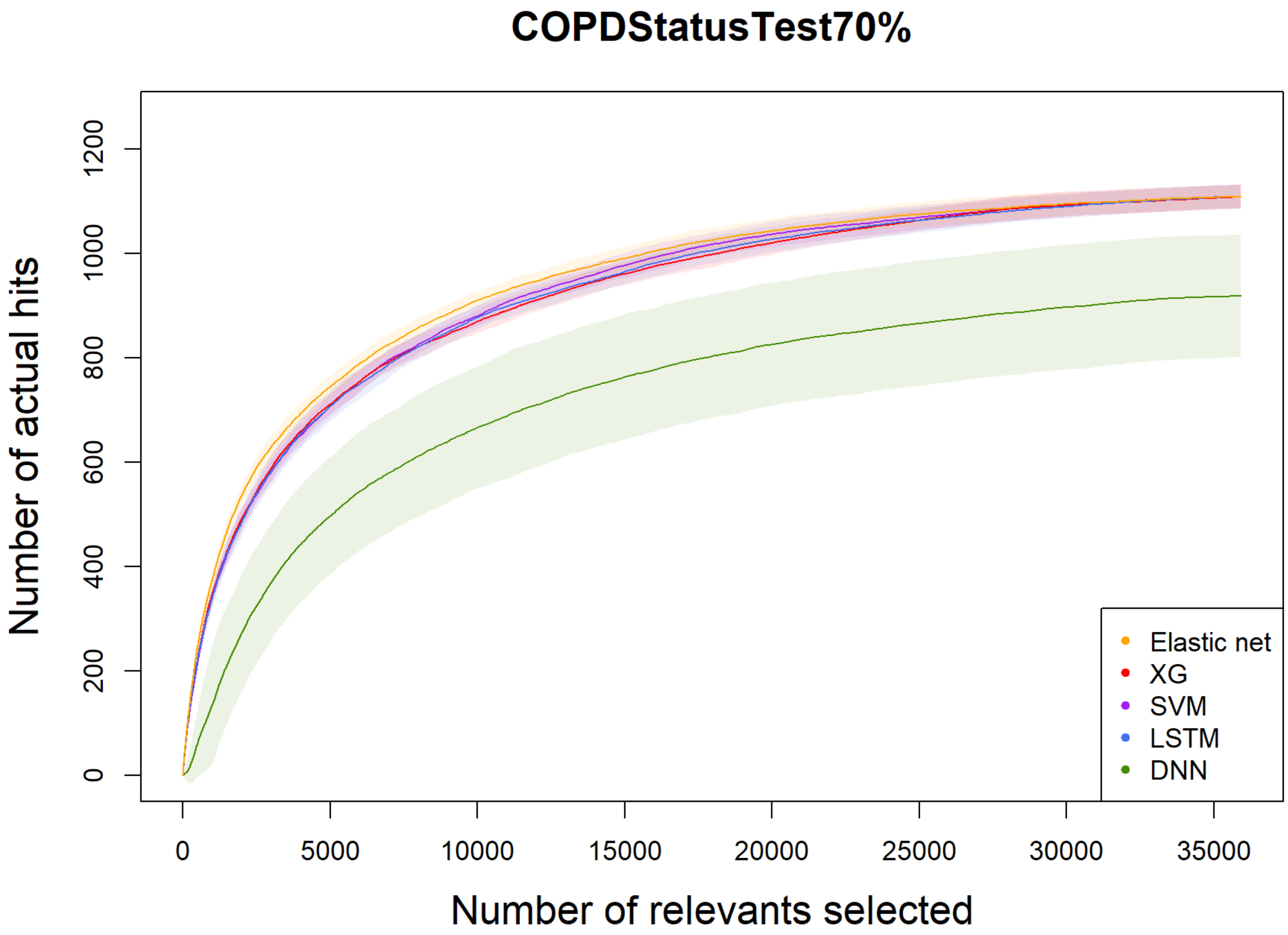

Figure S17. Hit curve on COPD for 70% dataset.

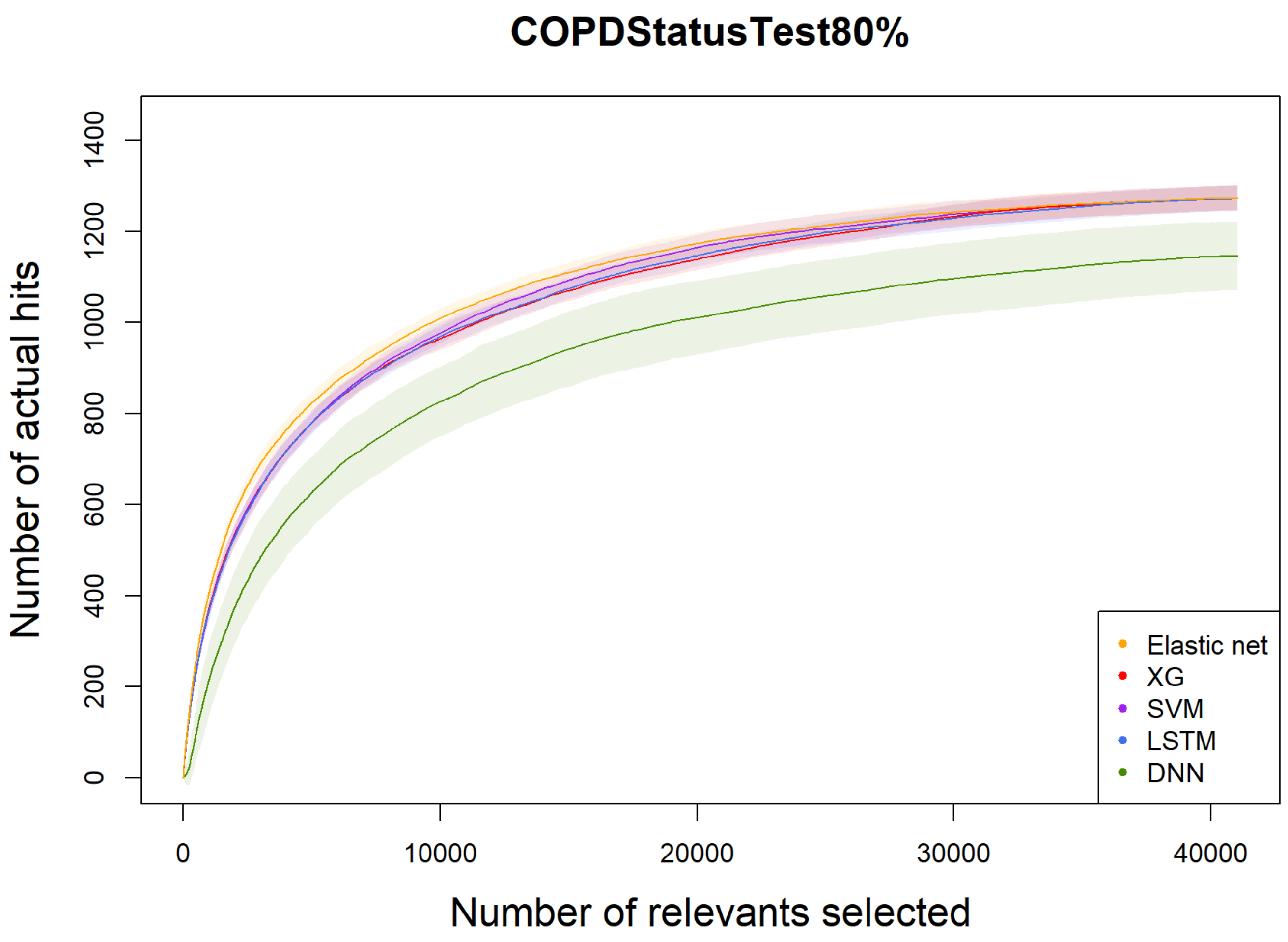

Figure S18. Hit curve on COPD for 80% dataset.

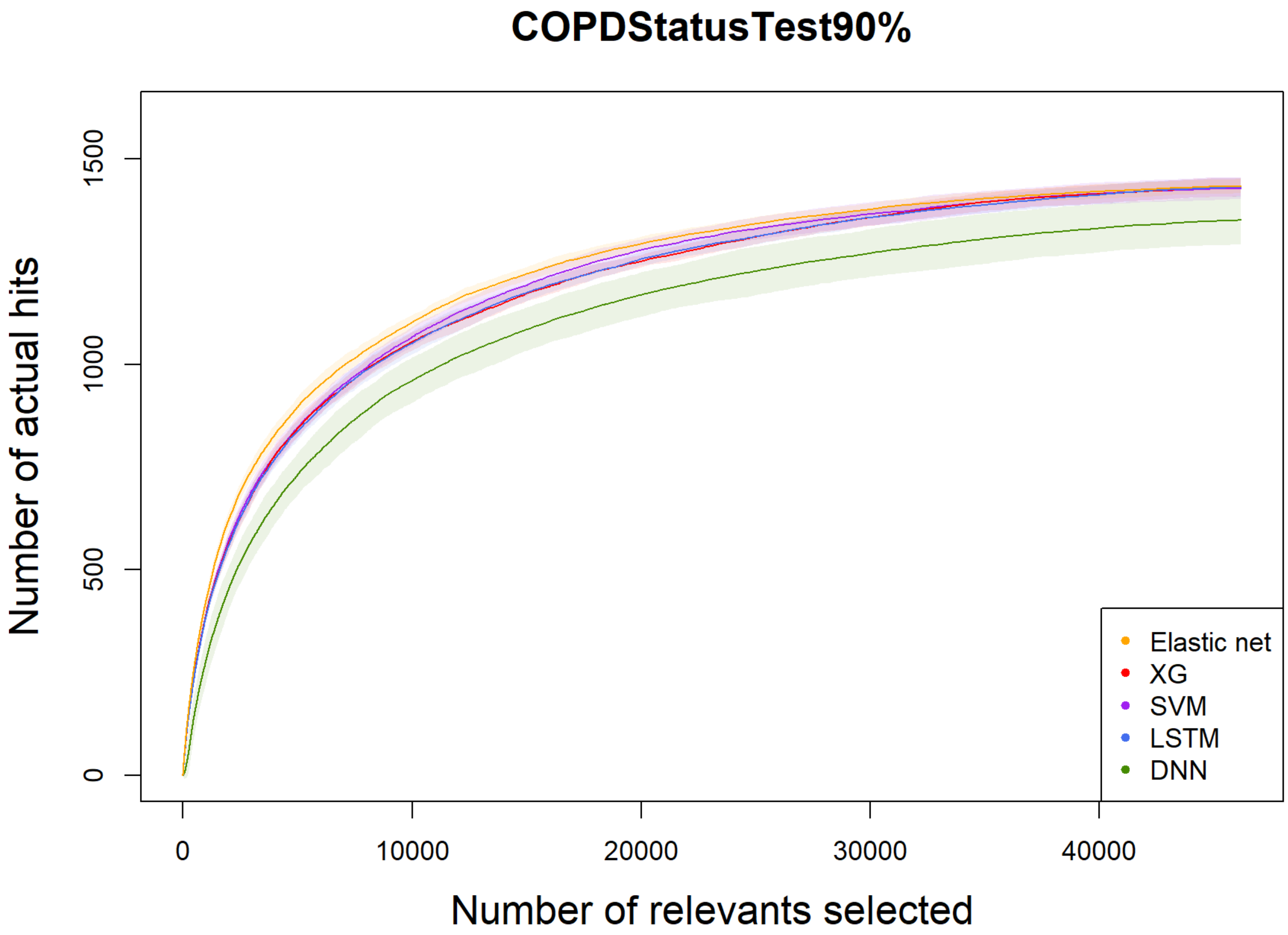

Figure S19. Hit curve on COPD for 90% dataset.

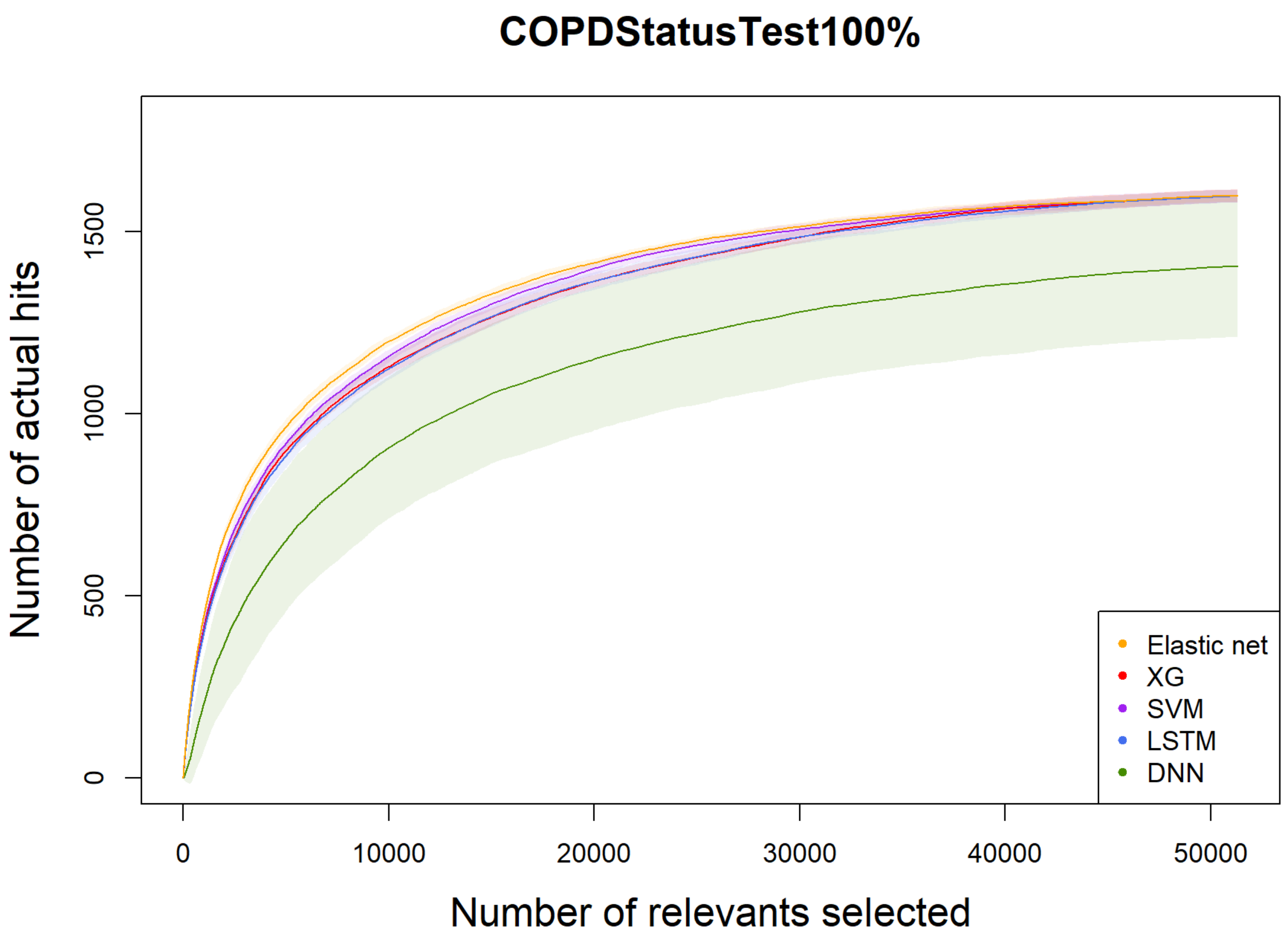

Figure S20. Hit curve on COPD for 100% dataset.

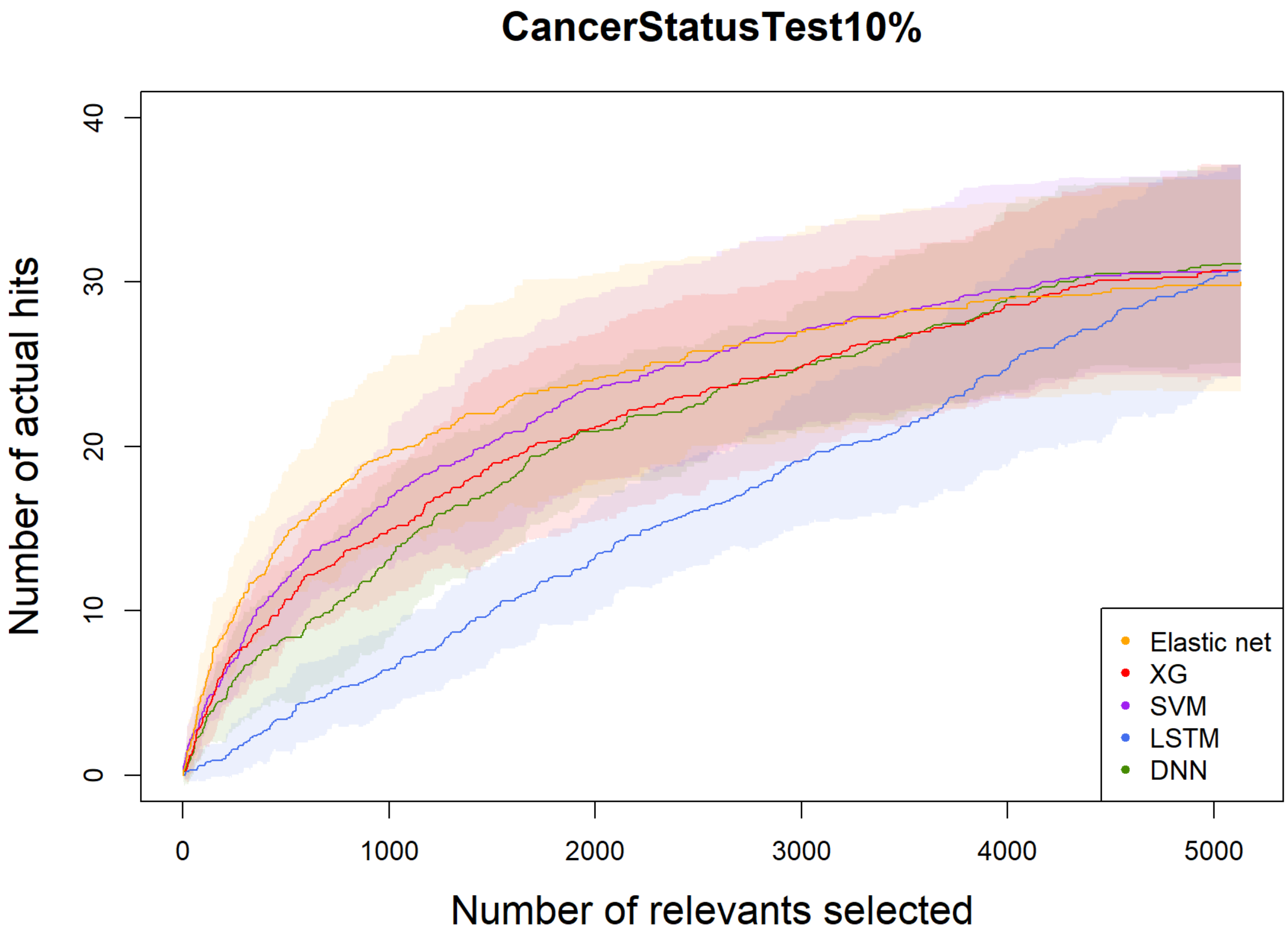

Figure S21. Hit curve on Cancer for 10% dataset.

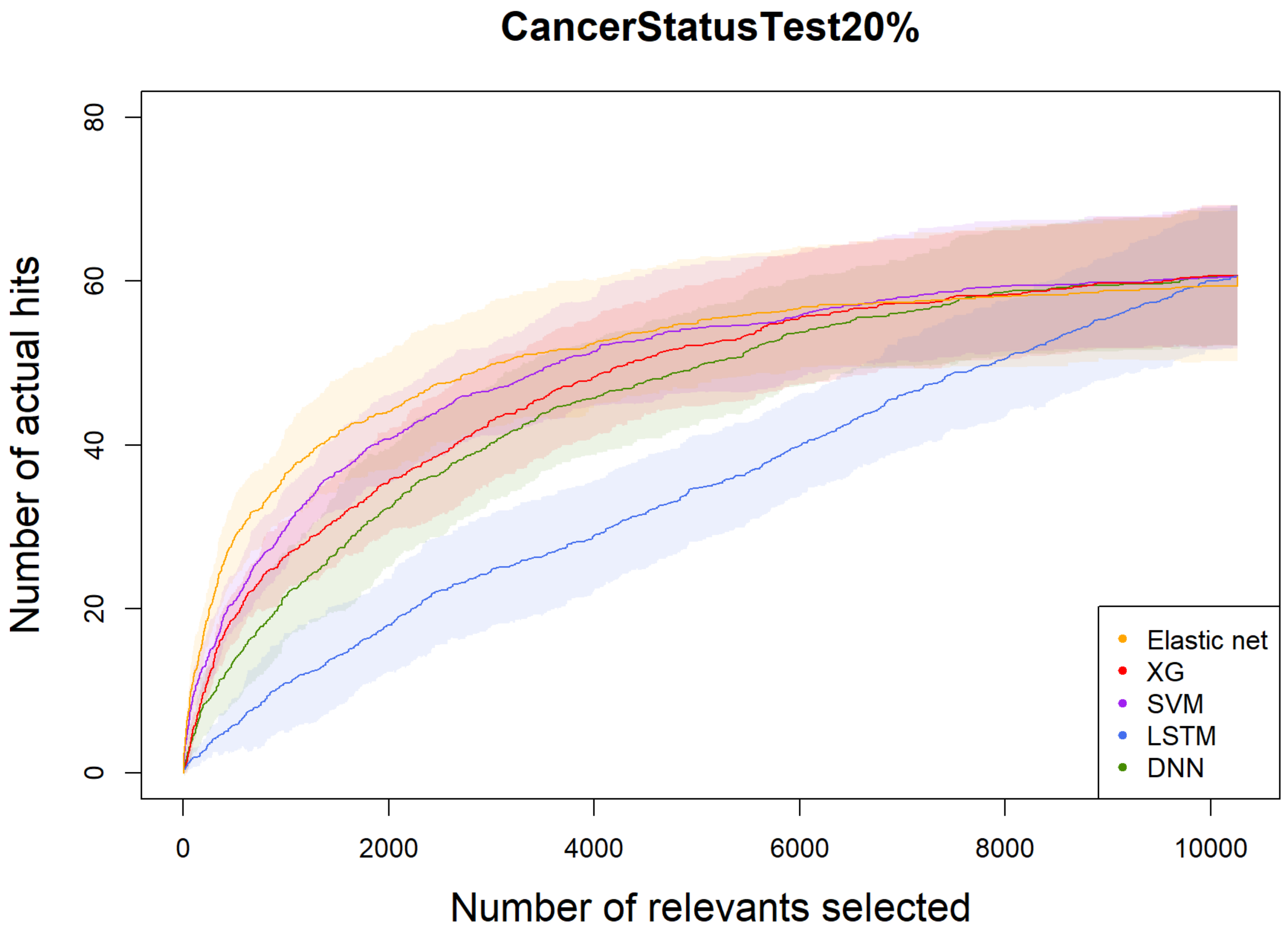

Figure S22. Hit curve on Cancer for 20% dataset.

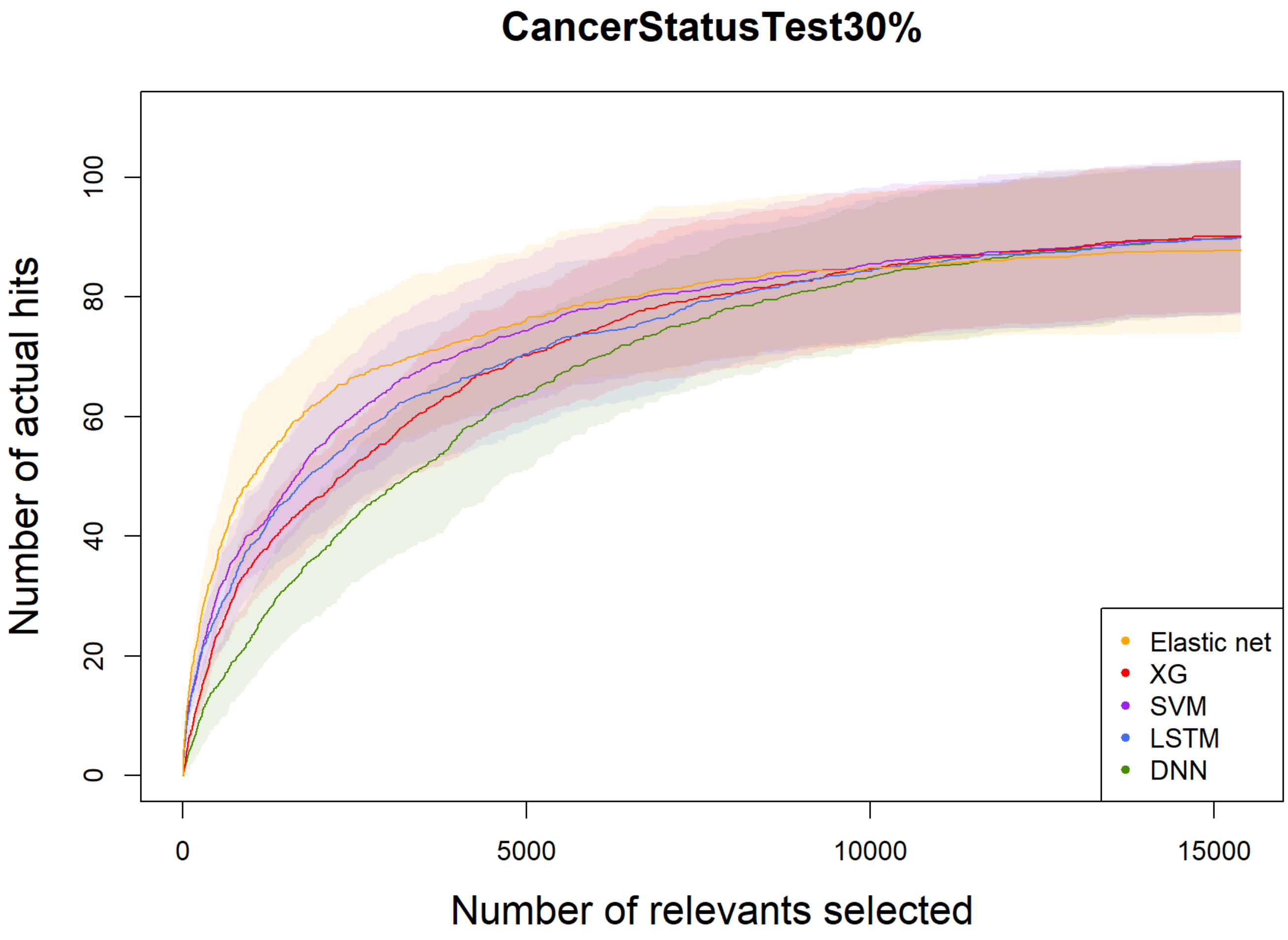

Figure S23. Hit curve on Cancer for 30% dataset.

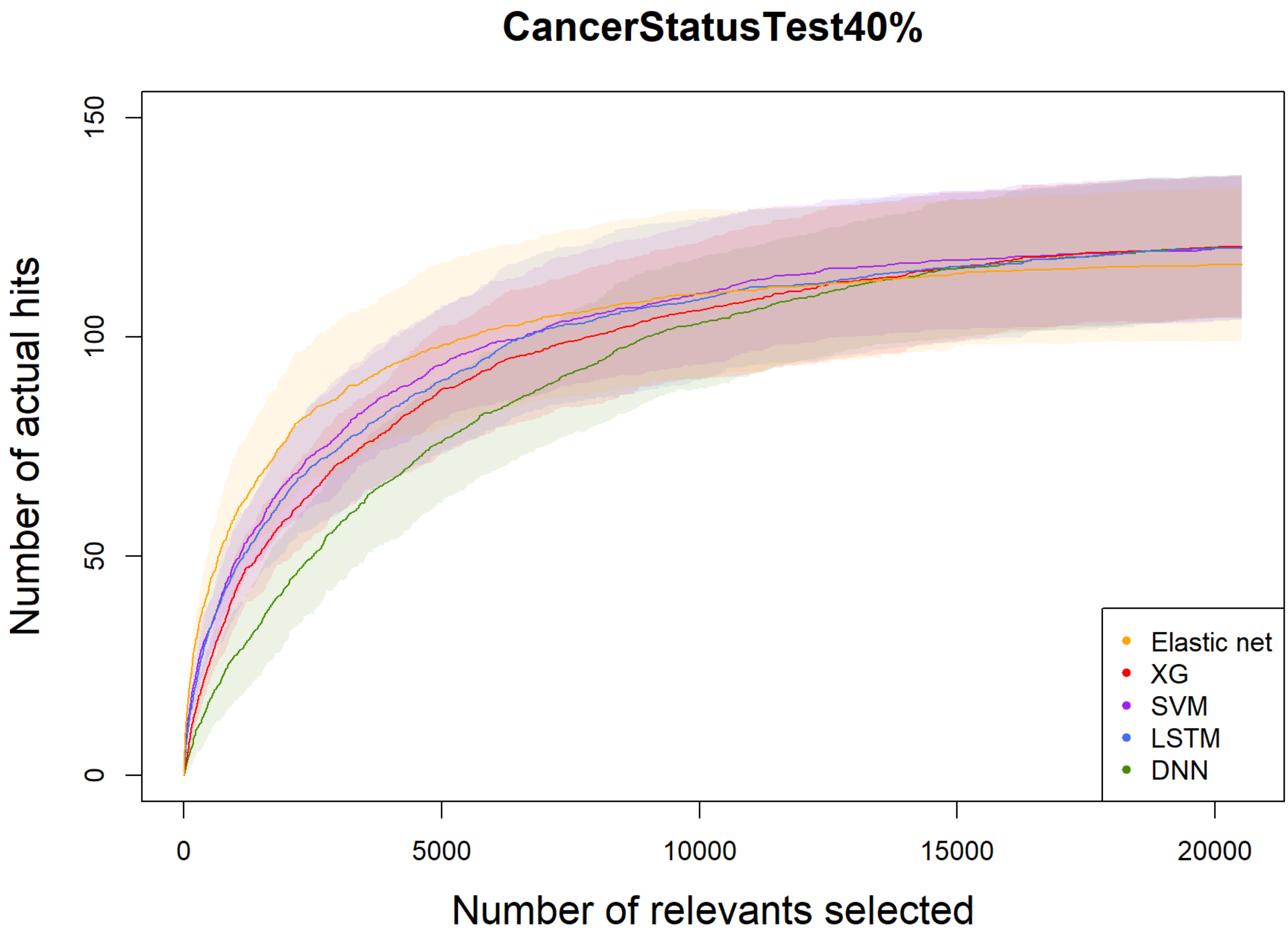

Figure S24. Hit curve on Cancer for 40% dataset.

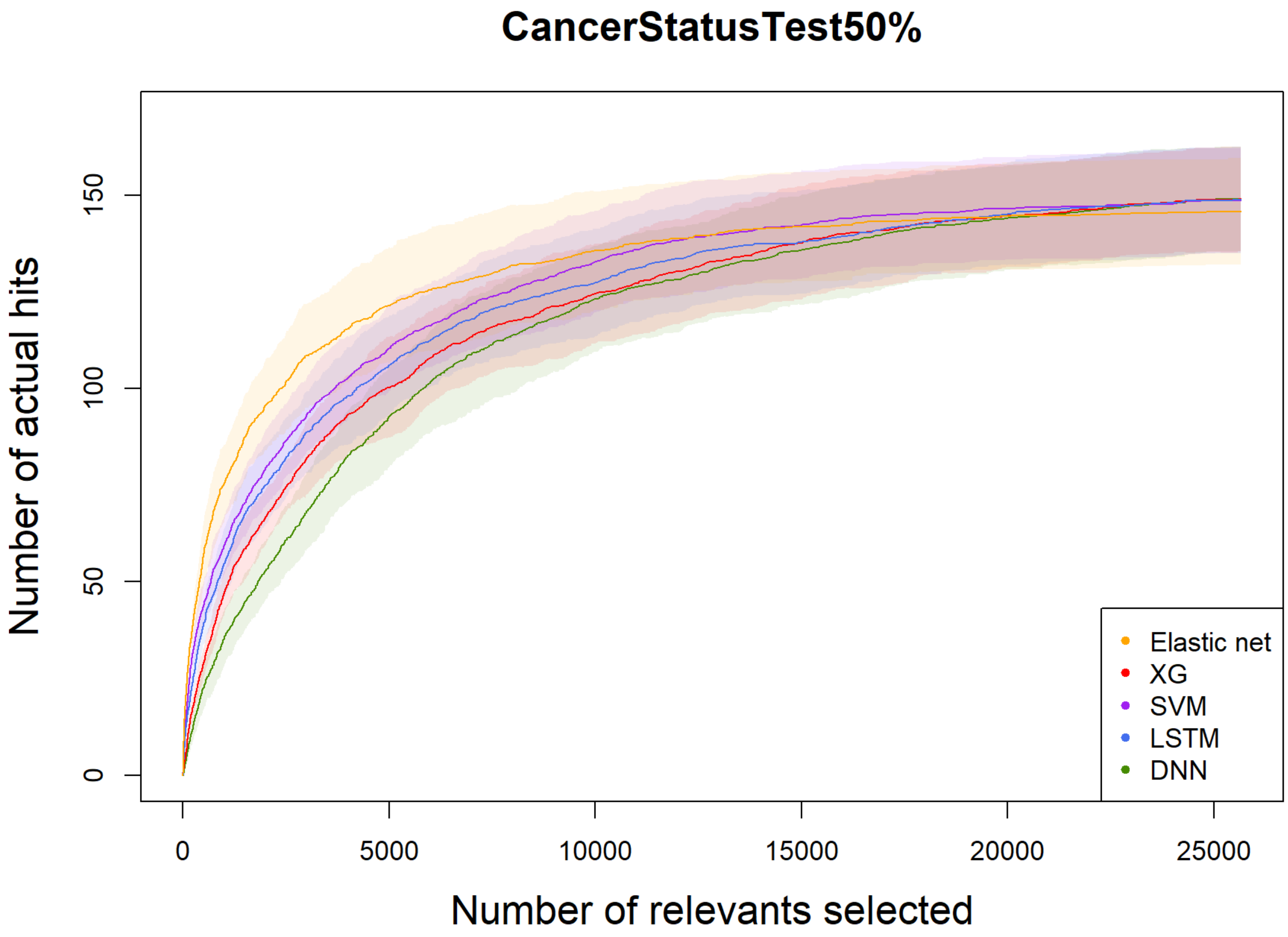

Figure S25. Hit curve on Cancer for 50% dataset.

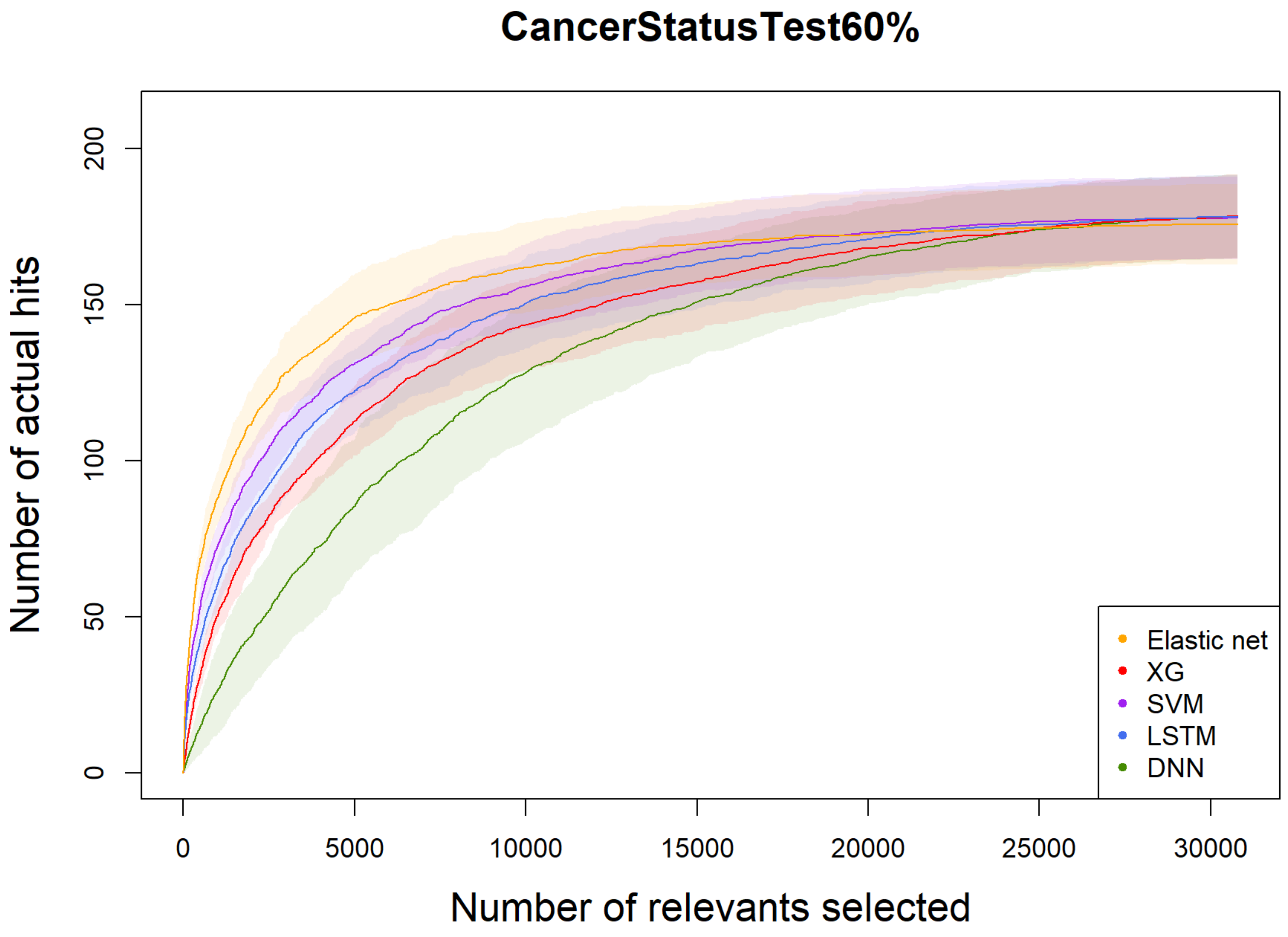

Figure S26. Hit curve on Cancer for 60% dataset.

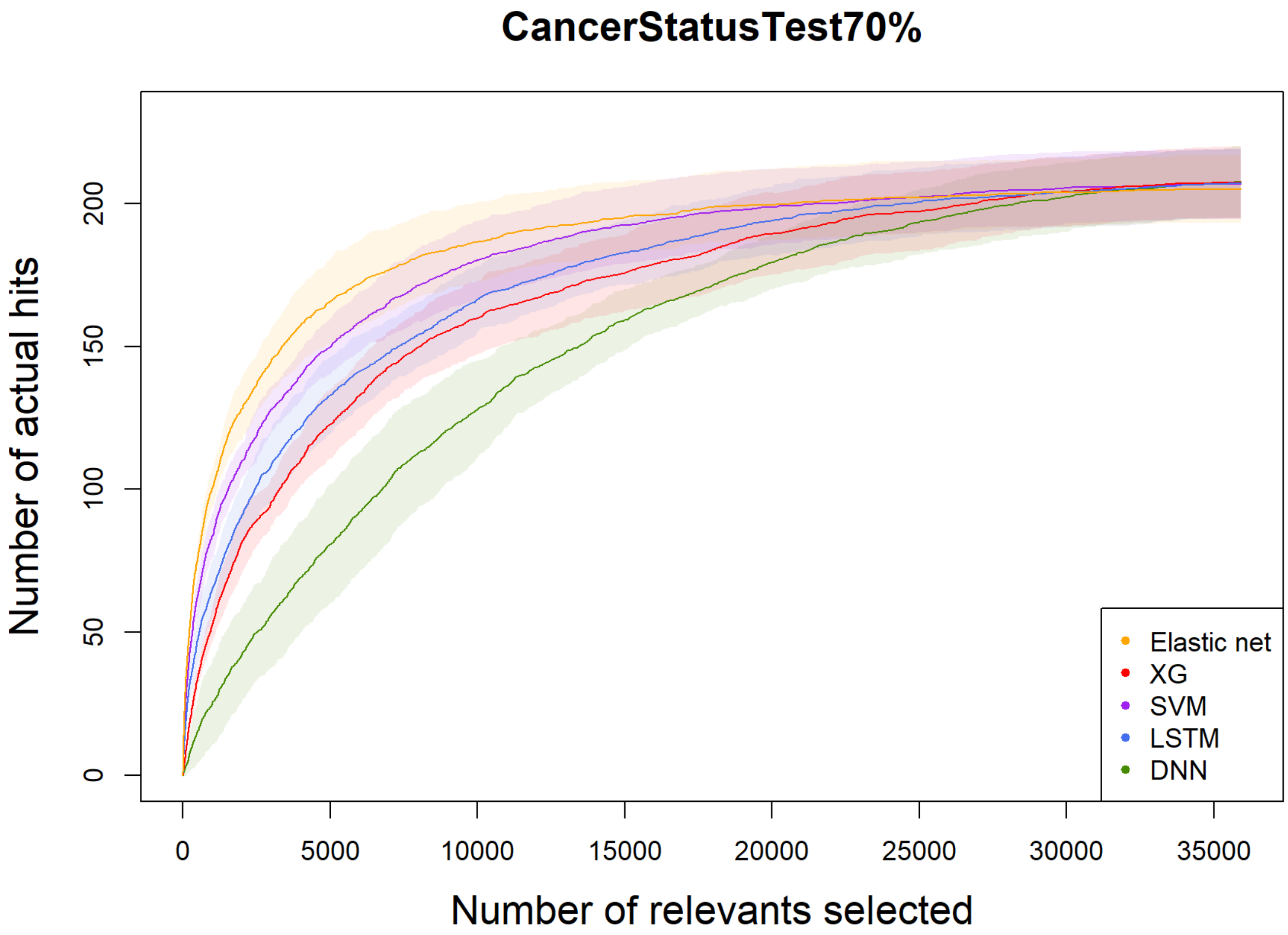

Figure S27. Hit curve on Cancer for 70% dataset.

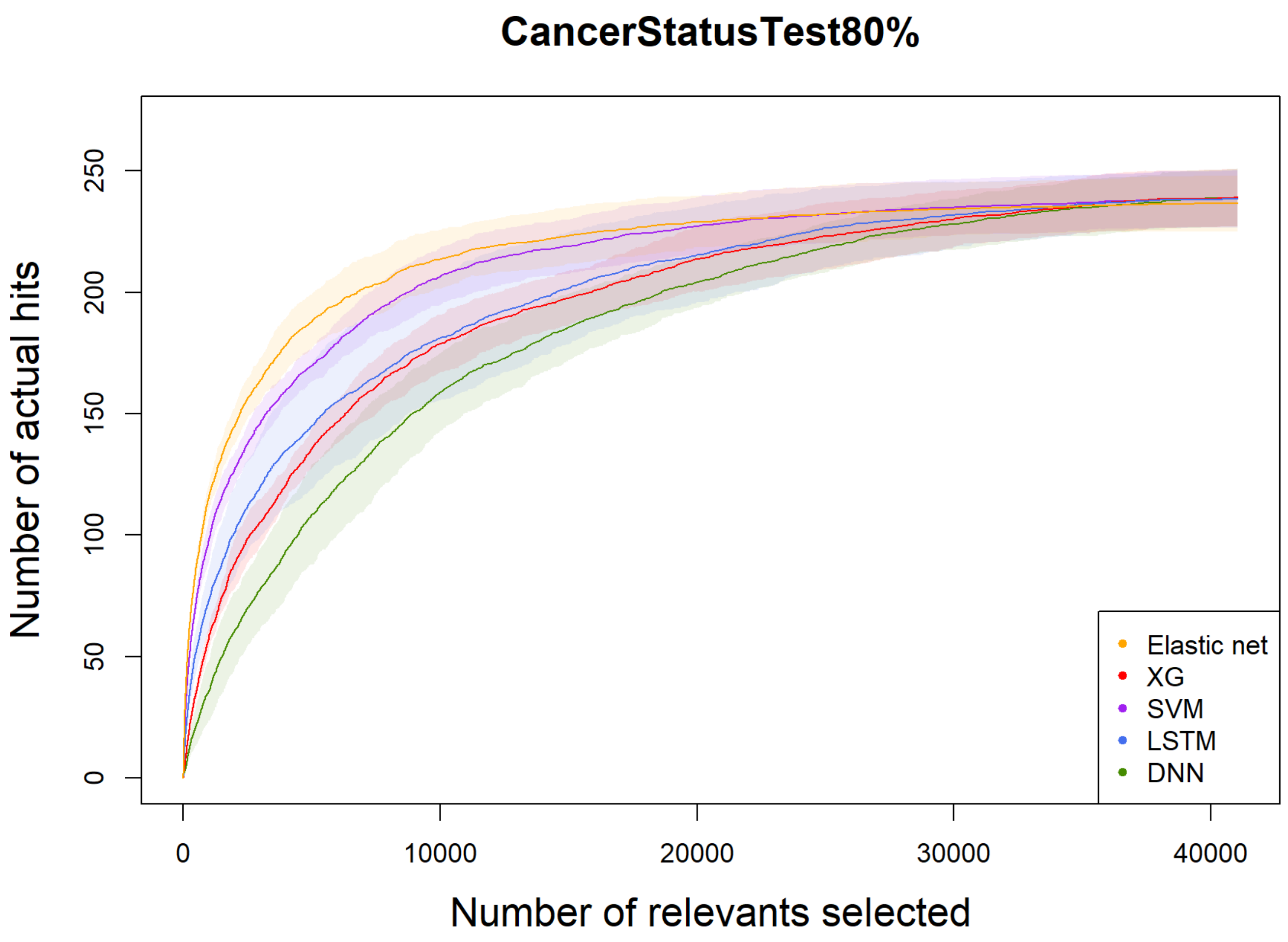

Figure S28. Hit curve on Cancer for 80% dataset.

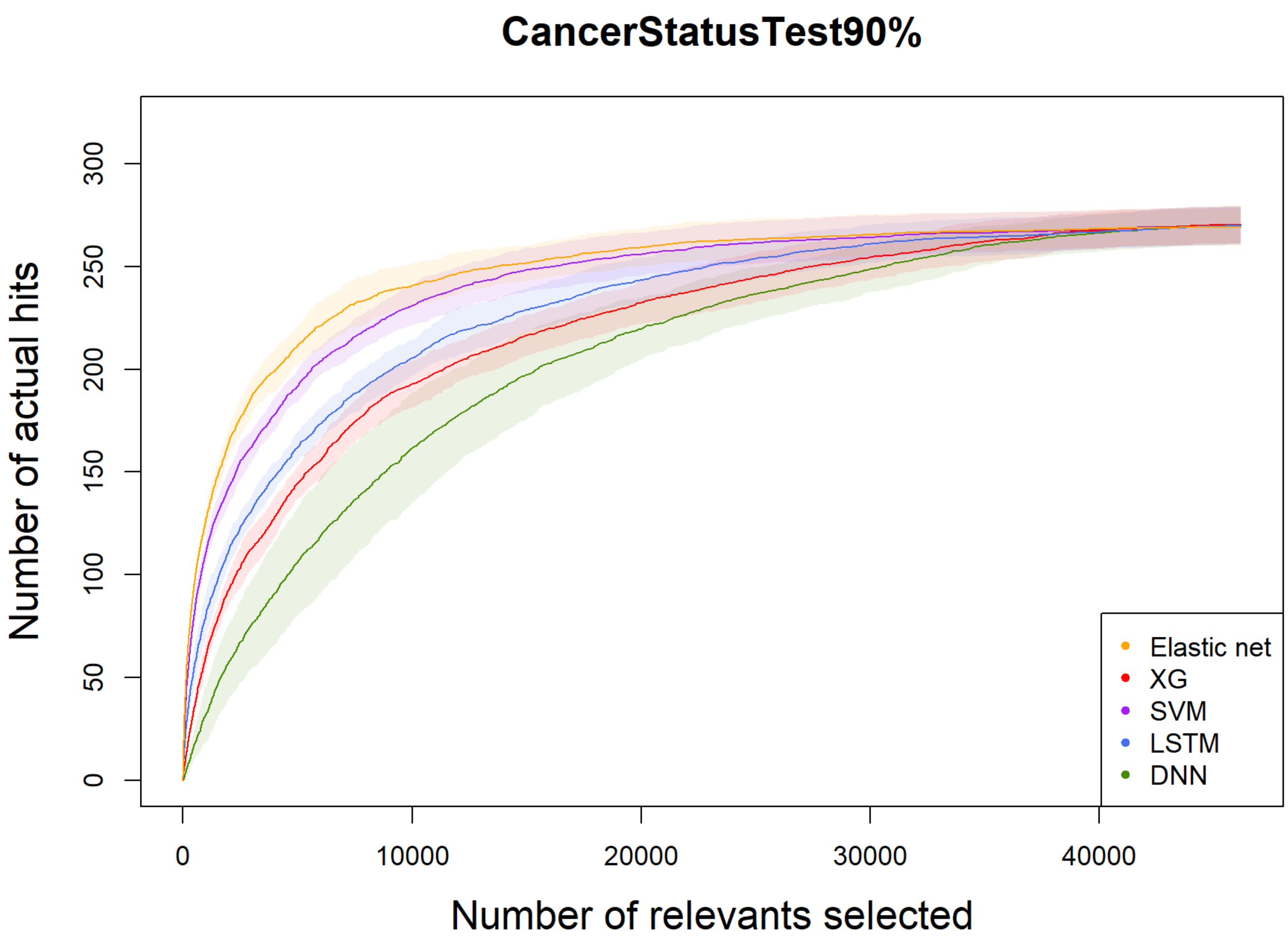

Figure S29. Hit curve on Cancer for 90% dataset.

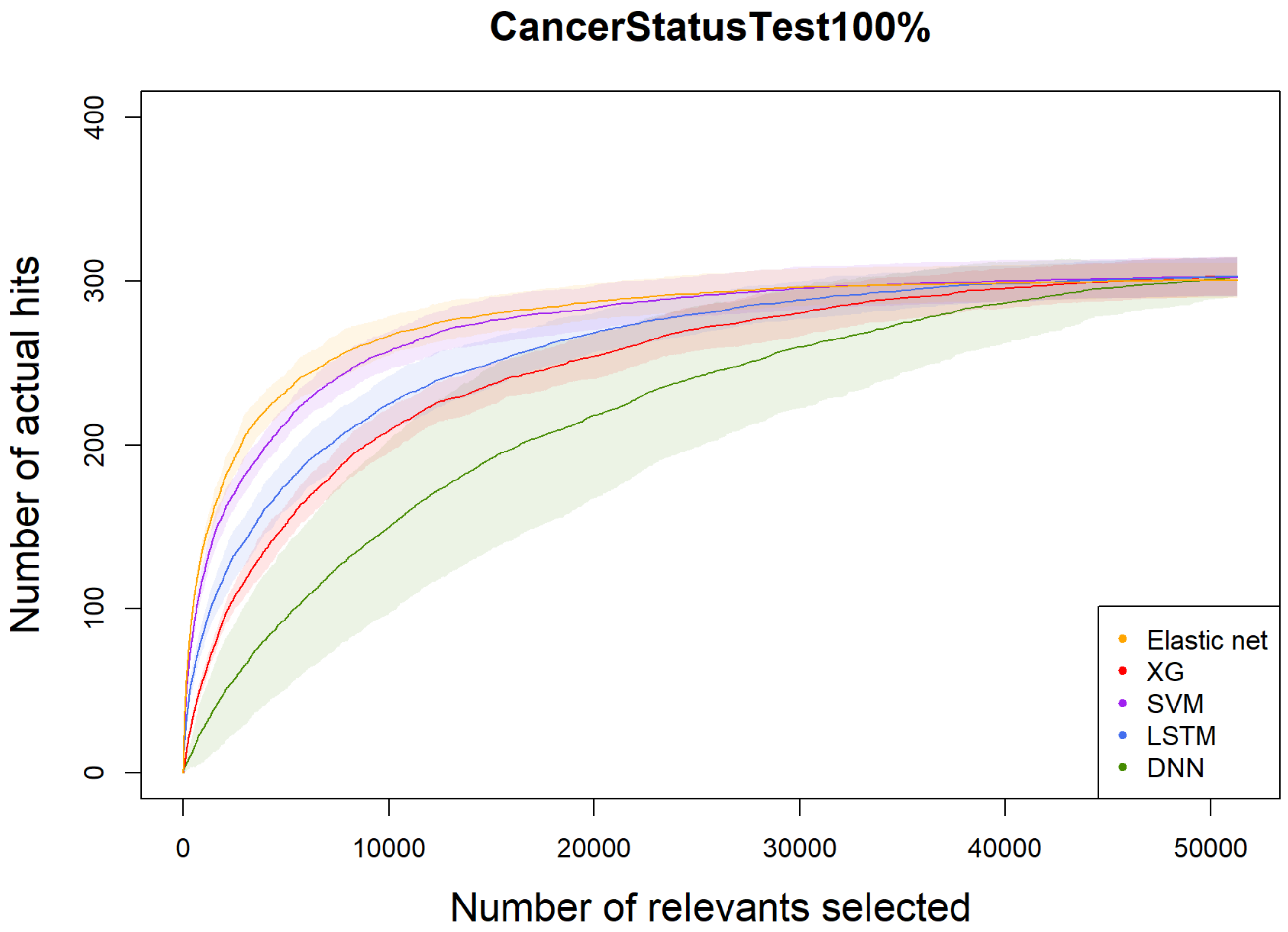

Figure S30. Hit curve on Cancer for 100% dataset.
